# Natural Compounds in Sex Hormone-Dependent Cancers: The Role of Triterpenes as Therapeutic Agents

**DOI:** 10.3389/fendo.2020.612396

**Published:** 2021-01-21

**Authors:** Codruţa Şoica, Mirela Voicu, Roxana Ghiulai, Cristina Dehelean, Roxana Racoviceanu, Cristina Trandafirescu, Oana-Janina Roșca, Gabriela Nistor, Marius Mioc, Alexandra Mioc

**Affiliations:** ^1^Faculty of Pharmacy, “Victor Babes” University of Medicine and Pharmacy, Timisoara, Romania; ^2^Department of Vascular Surgery, Pius Brinzeu Timisoara City Emergency Clinical Hospital, Timisoara, Romania

**Keywords:** phytocompounds, triterpenes, hormone-dependent cancers, antiproliferative activity, therapeutic agents

## Abstract

Sex hormone-dependent cancers currently contribute to the high number of cancer-related deaths worldwide. The study and elucidation of the molecular mechanisms underlying the progression of these tumors was a double-edged sword, leading to the expansion and development of new treatment options, with the cost of triggering more aggressive, therapy resistant relapses. The interaction of androgen, estrogen and progesterone hormones with specific receptors (AR, ER, PR) has emerged as a key player in the development and progression of breast, ovarian, prostate and endometrium cancers. Sex hormone-dependent cancers share a common and rather unique carcinogenesis mechanism involving the active role of endogenous and exogenous sex hormones to maintain high mitotic rates and increased cell proliferation thus increasing the probability of aberrant gene occurrence and accumulation highly correlated with abnormal cell division and the occurrence of malignant phenotypes. Cancer related hormone therapy has evolved, currently being associated with the blockade of other signaling pathways often associated with carcinogenesis and tumor progression in cancers, with promising results. However, despite the established developments, there are still several shortcomings to be addressed. Triterpenes are natural occurring secondary metabolites biosynthesized by various pathways starting from squalene cyclization. Due to their versatile therapeutic potential, including the extensively researched antiproliferative effect, these compounds are most definitely a cornerstone in the research and development of new natural/semisynthetic anticancer therapies. The present work thoroughly describes the ongoing research related to the antitumor activity of triterpenes in sex hormone-dependent cancers. Also, the current review highlights both the biological activity of various triterpenoid compounds and their featured mechanisms of action correlated with important chemical structural features.

## Introduction

Malignant diseases or cancers are the cause of a high number of deaths worldwide each year and are characterized by the abnormal, uncontrolled division of cells due to inherited or environmental genetic cellular changes. Cancer can be treated by multiple approaches such as surgery, radiotherapy or chemotherapy, all with severe side effects and the potential to induce tumor resistance; the use of plant-derived compounds may represent an appealing alternative due to their mild and well-tolerated biological effects and last but not least their affordable cost (1). Actually, plant-derived compounds were the starting point for numerous semisynthetic drugs or supplements currently used in cancer treatment; literature surveys reveal that only about one fifth of all commercially anticancer drugs are in fact obtained completely through chemical synthesis (2).

Sex hormone-dependent cancers affect both sexes and contribute to the high number of cancer-related deaths worldwide. In the last decade the deciphering of their underlying molecular mechanisms has expanded and improved treatment options thus leading to increased progression-free and overall survival. However, at the same time, the new treatment triggered more resistant and aggressive relapses (3). These types of cancer share the common feature of depending on male and female hormones, respectively, in order to continue to grow; therefore, estrogen and androgen receptors are essential for cancer development being the promoters of tumor growth. The biologic impact of sex steroid hormones is mediated by related receptors, which function as transcription factors in steroid responsive cells. The receptors often involved in mechanisms that lead to cancer progression are estrogen receptors (ER) and androgen receptors (AR). Estrogen receptors are encoded by two different genes and exist as two isoforms, namely ERα and ERβ. These two receptors have significantly different binding affinities for various substrates but both forms have high affinities for the endogenous estradiol. On the other hand, AR is activated, following the binding of two androgen hormone ligands, testosterone or 5α-dihydrotestosterone (4). Ligand binding to these steroid hormone receptors induces a set of cascade events (structural conformational changes, disassociation from heat shock protein, phosphorylation, dimerization) that leads to coregulatory molecules recruitment. These coregulatory factors bind to response elements found in the promotor region of specific genes, leading to gene transcription (5). The expression of these coregulator molecules is presumably a significant determinant of steroidal hormone stimulation tumor response. Ligand-mediated hormone receptor activation is highly regulated at many levels (transcriptional, posttranscriptional, post-translational) as revealed by microRNAs activity (6). In cancer cells, the normal regulatory mechanisms controlling steroid hormone-induced receptor activation can be disrupted, leading to a balanced disruption between hormone levels and proliferation. While the receptors are most responsive to their high affinity ligands, when the most favorable hormone is depleted, binding of alternative lower affinity steroids may initiate transcription thereby contributing to the progression of disease (4).

Sex hormone-dependent cancers involve cancers of the breast, endometrium, ovary, prostate and testis and they all share a common and rather unique carcinogenesis mechanism; while in other cancers viruses and chemical agents are considered tumor initiators and promoters, in sex hormone-dependent cancers endogenous and exogenous sex hormones maintain cell proliferation thus increasing the probability of random genetic errors, which may finally result in abnormal cell division and the occurrence of malignant phenotypes (7). In addition, the hormonal stimulation of cell proliferation continues during tumor growth.

**Figure 1:**
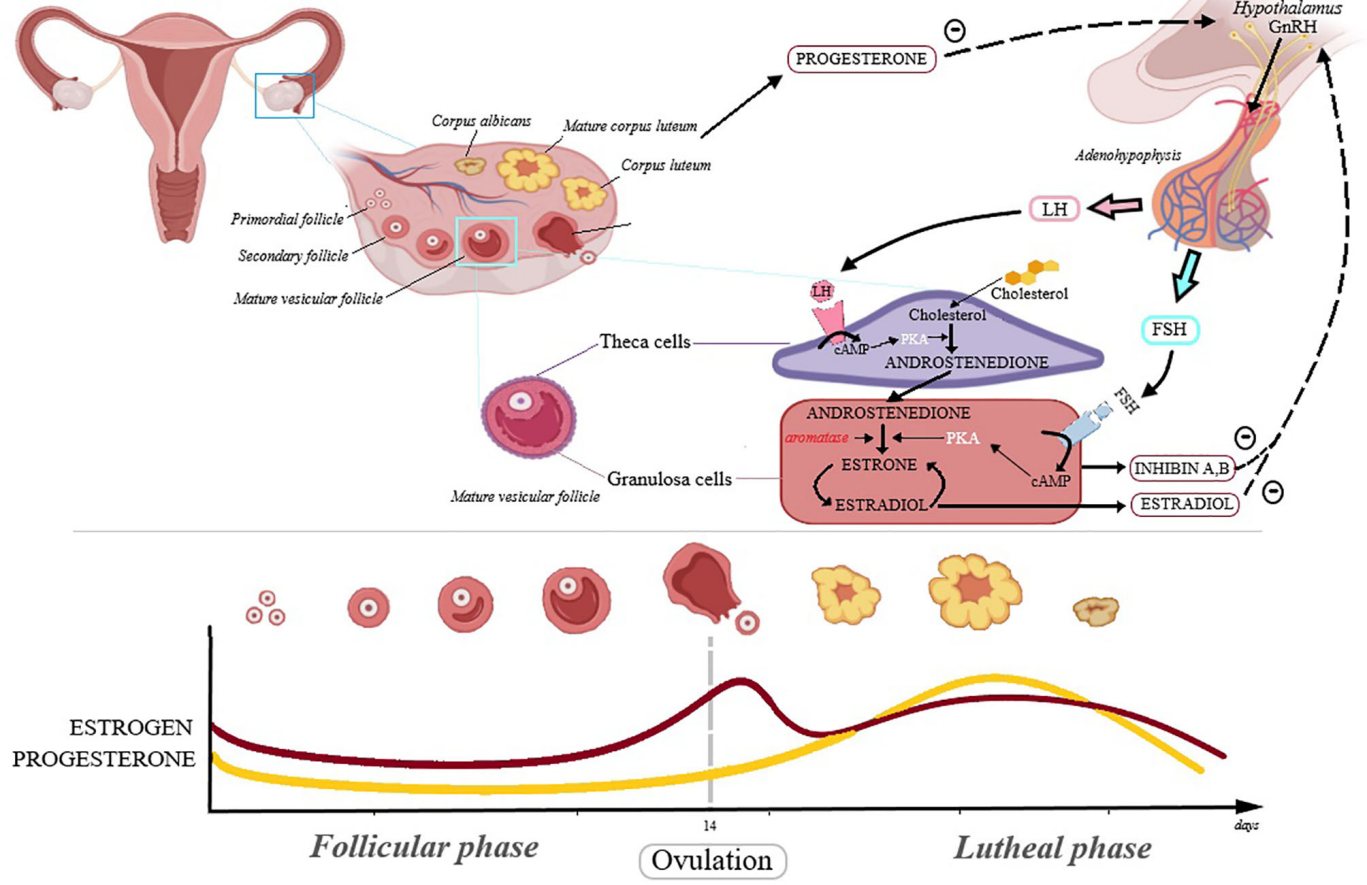
The hormonal interaction at ovary level. In the first phase of the menstrual cycle, called follicular phase, the follicle-stimulating hormone (FSH) concentration is increasing as follicles maturate, reaching a maximum during ovulation. FSH binds to the FSH receptor in the granulosa cells influencing androstenedione conversion to estradiol. The luteinizing hormone (LH) triggers ovulation and its levels are maximum during this phase. LH binds to the LH receptors from theca cells and promotes conversion of cholesterol into androstenedione. Inhibin A and B are members of transforming growth factor beta (TGFB) family and regulate FSH activity. The corpus luteum is formed after ovulation and secrets progesterone. During most of the menstrual cycle, the neuroendocrine output (from hypothalamus and adenohypophysis) is modulated by negative feed-back from progesterone and estrogen.

Ovarian cancer is the fifth leading cancer-related cause of death in women and is considered the most severe gynecological malignancy; it shows no specific symptoms, which combined with the lack of early detection methods and yet unclear etiology lead to delayed diagnosis and poor prognosis (8). Reproductive hormones play an essential role in the development of ovarian cancer, confirmed by the finding that the continuous administration of oral contraceptives leads to a lower risk of ovarian cancer, presumably due to reduced levels of endogenous estrogens, as a result of negative feedback regulation (8); the hormonal interaction at ovary level are depicted in **Figure 1**.

**Figure 2:**
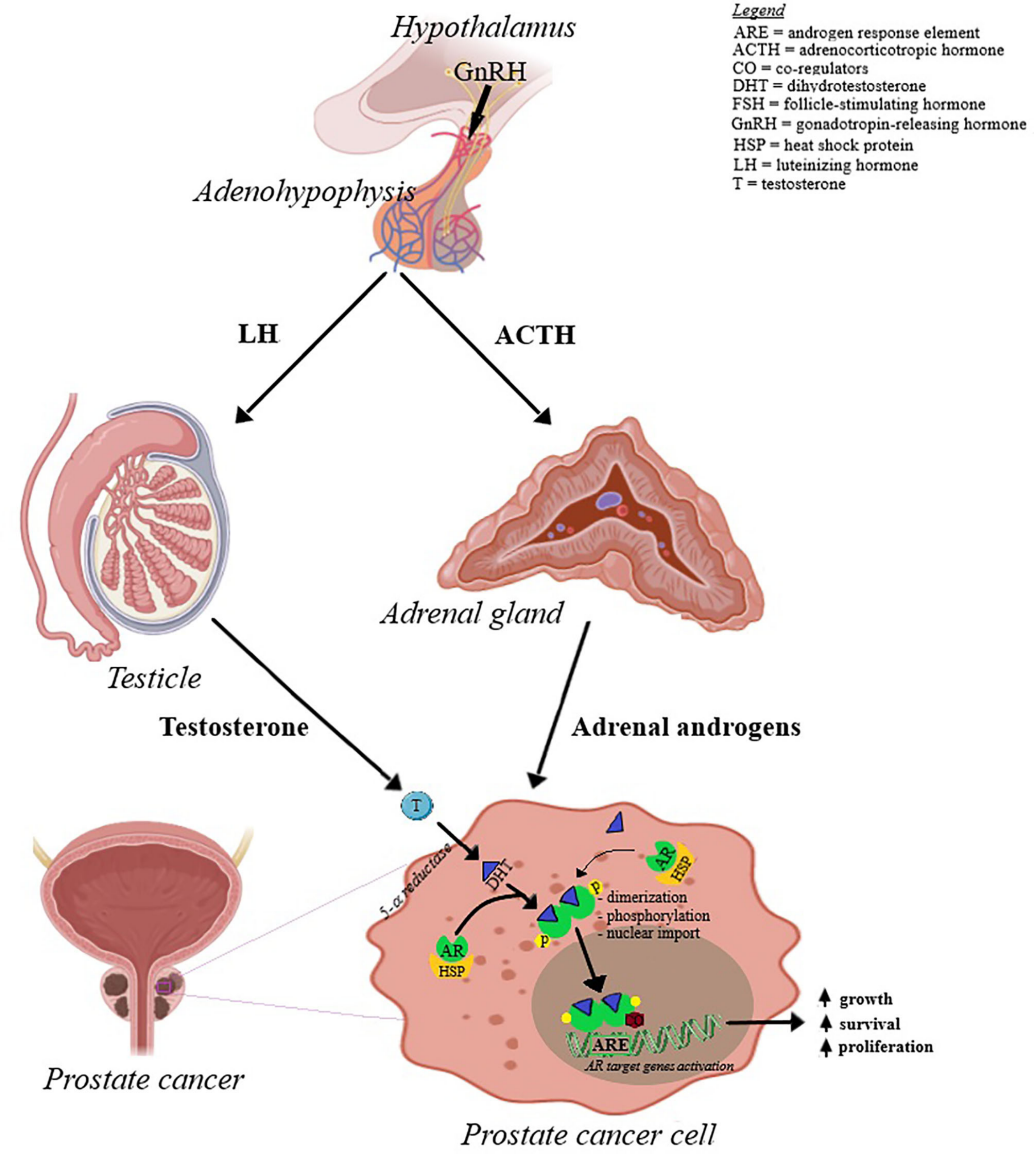
The mechanisms involved in prostate cancer development. Androgens are produced by the adrenal glands and testicles under the influence of adrenocorticotropic hormone (ACTH) and luteinizing hormone (LH), respectively. ACTH and LH release from adenohypophysis are under the control of hypothalamic gonadotropin-releasing hormone (GnRH). Androgens, mainly testosterone, is converted in dihydrotestosterone (DHT) that enters prostate cancer cell. The inactive form of androgen receptor (AR) resides in the cell cytoplasm, due to its binding to heat shock protein 90 (HSP). AR binds to the dihydrotestosterone (DHT) and dissociates from HSP. Ligand-bound AR then suffers dimerization, phosphorylation by MAPK and is translocated to the nucleus by an intrinsic nuclear localization signal. At the nuclear level, AR interacts with the androgen response element (ARE) and controls target gene expression.

**Figure 3:**
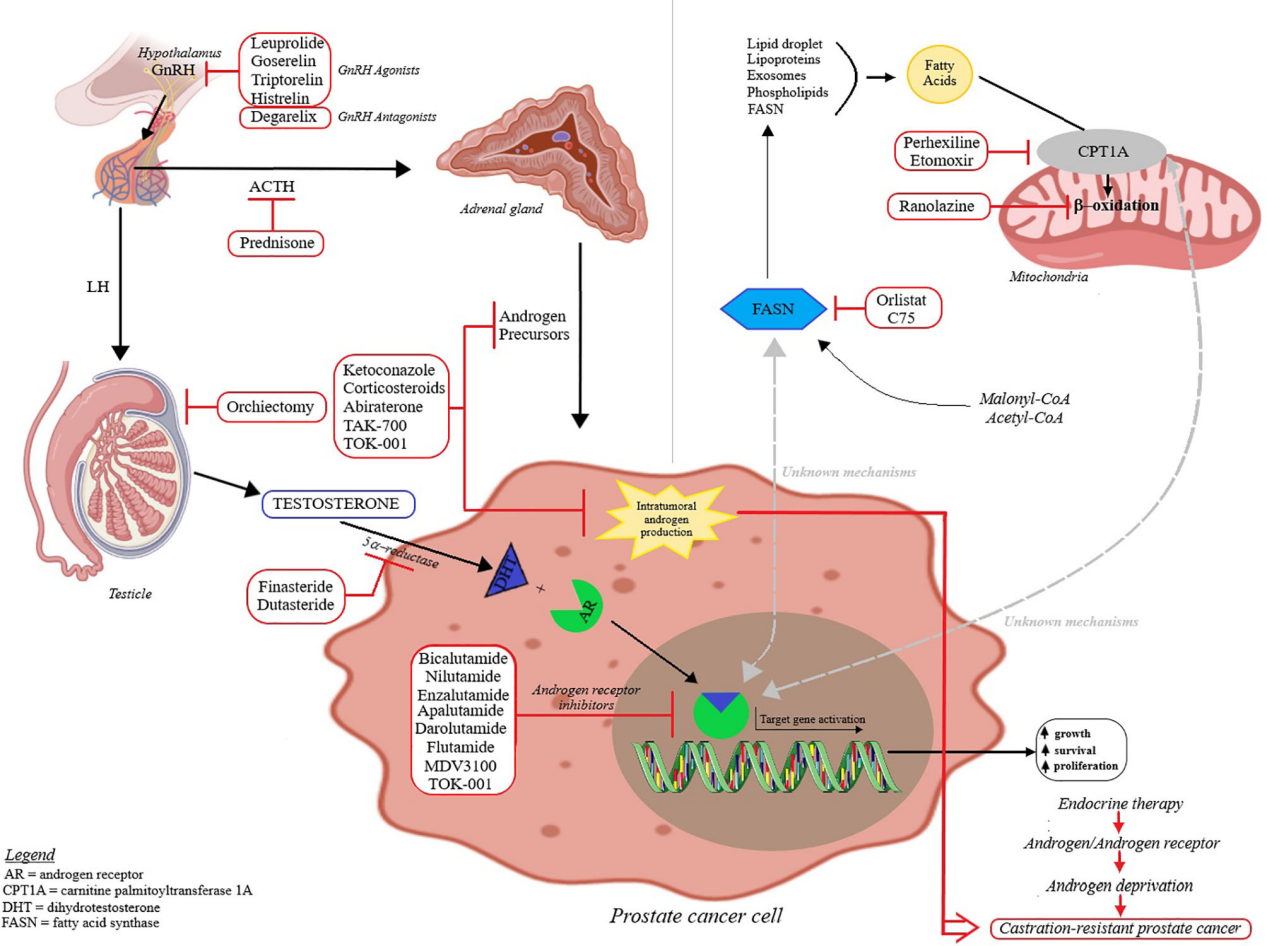
The endocrine therapy in prostate cancer and approved therapies – the mechanism of action of androgen deprivation therapy and lipid metabolism involvement in prostate cancer. The majority of the total available testosterone (90-95%) is produced by the testicles and the reminder by the adrenal glands. Testosterone is converted by 5α-reductase to dihydrotestosterone (DHT) that binds inside the cell to the androgen receptor (AR) and translocate to the nucleus. Upon binding to the androgen response element, it acts as a transcription factor and signals downstream targets. Androgens are known to regulate the activity of carnitine palmitoyltransferase 1A (CPT1A) and fatty acid synthase (FASN); the balance between fat synthesis and oxidation is also modulated by the tumor environment. A better understanding of this tumor dependency can improve the potency of anticancer agents that target lipid metabolism and can be used in combination with androgen deprivation therapy.

Prostate cancer is cited as one of the most common cancer-related cause of death in men; its occurrence and development is primarily dependent on androgen binding as well as transcription signals of the specific androgen receptors (9, 10). Androgens play a key role in cellular growth of both normal and pathological prostate tissue (11); the presumed mechanisms of prostate cancer development are depicted in **Figure 2**. Based on the identified hormone-related tumor growth, the first treatments consisted in androgen-deprivation therapy. Currently, the therapeutical options have evolved by associating chemotherapy, radiotherapy and immunotherapy, depending on the tumor, patient or drug side effects (12–14). The challenges encountered by the endocrine therapy in prostate cancer are depicted in **Figure 3**; one important finding is that a Western diet rich in long chain fatty acids may promote prostate cancer to lethal stages (15).

**Figure 4:**
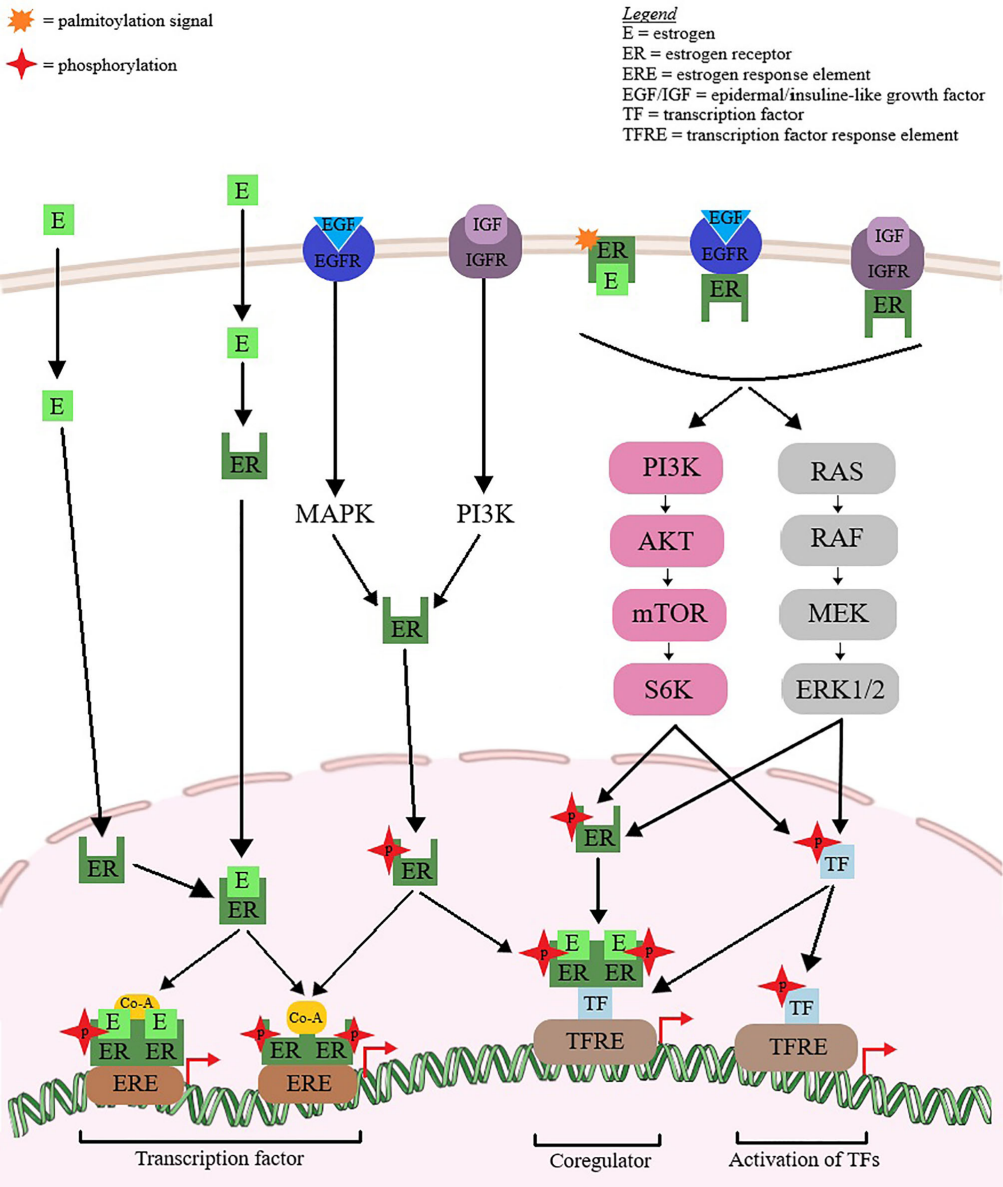
Hormone-dependent breast cancer progression *via* estrogen-mediated signaling pathways. Estrogen (E) binds to the estrogen receptor (ER). The ligand binding induces ER dimerization, phosphorylation and nuclear translocation to the ERE region. Coactivators are recruited and the complex affects gene transcription. ER can act as a coregulator for some transcription factors. ER phosphorylation and nuclear activity can also be produced by growth factors (EGF/IGF). The extranuclear ER can activate some transcription factor by interfering with PI3K, ERK1/2 and MAPK signaling pathways.

Breast cancer etiology is strongly dependent on reproductive hormones, in particular estrogens (16), both endogenous and exogenous, as exposure to such hormones has been proven to be directly linked to increased risk of breast cancer (17, 18); hormone receptor-positive types of breast cancer are clinically more responsive to hormonal treatment compared to hormone receptor-negative tumors. Therefore, estrogen and/or progesterone receptor-positive breast cancer patients exhibit better prognosis and lower mortality risks compared to women with negative disease (19). In addition, the estrogen receptor (ER) serves as prognostic marker for tumor responsiveness to endocrine therapeutic approaches (20, 21). Recent studies have identified estrogen as a potential immune regulatory agent in the tumor microenvironment, able to modulate the tumor response without acting directly on tumor cells (22). The signaling pathways through which estrogens promote the progression of hormone-dependent breast cancer are depicted in **Figure 4**.

**Figure 5:**
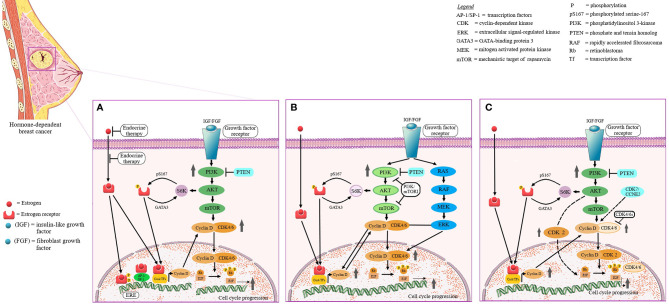
The identified signaling pathways involved in the mediation of sensitivity/resistance versus endocrine therapy. The signaling pathways that involve cyclin D—cyclin-dependent kinase 4 and 6 (CDK4/6)/6-INK4- retinoblastoma (Rb) and phosphatidylinositol-3 kinase/mechanistic target of rapamycin (PI3K/mTOR) play a central role in cell cycle progression, cellular differentiation, growth, survival and metabolism. Dysregulation of these pathways results in genetic/epigenetic alterations, increased proliferation and evasion of apoptosis, as observed in hormone receptor-positive breast cancer (HR)+, and have been involved in the development of endocrine therapy resistance. PI3K/mTOR pathway activates S6 kinase and leads to estrogen receptor (ER) signaling activation. Activation of ER pathway upregulates the protein expression of cyclin D, activates cyclin D—CDK4/6/6-INK4- Rb pathway and increases cell cycle progression **(A)**. The resistance to PI3K/mTOR pathway inhibitors occurs *via* an aberrant activation of RAS/RAF/MEK/ERK pathway that produces an additional assembly of cyclin D-CDK4/6 complexes **(B)**. In HR+ breast cancer the cyclin D - CDK4/6/6-INK4- Rb pathway, in particular cyclin D and CDK4, is overexpressed. Activation of PI3K/mTOR pathway leads to cyclin D upregulation, binding and activation of CDK2 (if CDK4/6 is absent) and consecutive Rb phosphorylation and cell cycle progression. CDK4/6 inhibitors have synergistic effects to endocrine therapy **(C)**.

The endocrine therapy represented an important tool in breast cancer treatment ever since the discovery of the role of ovarian hormones in tumor growth (23) and has continuously improved due to the more and more profound understanding of the underlying molecular mechanisms which regulate tumor sensitivity to hormones; subsequently, it has produced huge progress in terms of prognosis of hormone receptor-positive breast cancers (24). Currently, CKD4/6 inhibitors, mTOR (mammalian target of rapamycin) inhibitors and PI3K (phosphatidylinositol-3 kinase) inhibitors are investigated in order to overcome tumor resistance occurred through genetic and molecular changes (25). The identified signaling pathways involved in the mediation of sensitivity/resistance versus endocrine therapy are depicted in **Figure 5**. The most promising therapeutic option consists in the triplet combination-based endocrine therapy which associates PI3K or mTOR inhibitors to combined CDK4/6 and endocrine inhibitors in order to prolong CDK4/6 inhibitor sensitivity (26). Moreover, recent studies have highlighted the fact that common signaling pathways are shared by sex hormones. In this regard, an early estrogen-responsive gene, GREB1, was identified which responds to both estrogen and androgen levels and is able to act as a pan-steroid responsive gene, stimulating the proliferation of breast, ovarian and prostate cancer cells (27); GREB1 may serve as clinical marker to monitor the response to endocrine therapy but also as a potential therapeutic target (28).

The present review aimed to deliver updated information about the *in vitro* and *in vivo* studies regarding the anticancer effect of both crude and triterpene-enriched extracts as well as isolated triterpenoids from natural sources in reproductive hormones-dependent cancers. The data was collected from three major databases, that are worldwide recognized by the scientific community: PubMed (http://www.ncbi.nlm.nih.gov/pubmed), Google scholar (http://scholar.google.com) and Web of Science (WOS, www.webofknowledge.com). The databases were searched selecting the option “all fields” in the query box and using combinations of the following keywords: pentacyclic triterpenes, tetracyclic triterpenes, hormone-dependent cancer, breast cancer, prostate cancer, cervical cancer, ovarian cancer. The articles were sorted by “best match” criteria and included articles published over the last 2 decades, from 2000 to present. The inclusion criteria upon which the manuscript selection was based included: articles that had the keywords in the title, articles that studied crude, triterpene-enriched extracts, or isolated triterpenoids from natural sources in breast, prostate, ovarian, and cervical cancer, abstract or full-text. The exclusion criteria included duplicate articles, articles with irrelevant, ambiguous and incomplete information, articles published in other language than English and articles with not of interest outcomes. The screening of all databases identified 2,842 results; however, after the application of inclusion/exclusion criteria, only 199 were subjected for further review.

## Triterpenes as Biologically-Active Agents

Triterpenes are secondary metabolites found particularly in dicotyledonous plants and resulted from squalene cyclization by various chemical pathways (29). Most triterpenoids consist of 30 carbon atoms organized in six isoprene rings and can be classified as tetra or pentacyclic compounds (30). The literature cites numerous reviews on the topic of triterpenes and their saponins thus showing the high interest this chemical class raised among researchers, interest which is based on their wide spread in plants as well as their large plethora of biological activities, including antitumor and anti-inflammatory. So far, approximately 30,000 triterpenic compounds have been identified and classified according to their chemical structure and properties; in terms of biological significance, the most important representatives are the pentacyclic oleanane-, ursane-, and lupane-derivatives (31) and the tetracyclic dammarane- and euphane-derivatives (32). In addition to their anticancer, chemopreventive and chemo sensitizing effects, triterpenes can be used as organ protective agents against drug induced toxicities (33).

**Figure 6:**
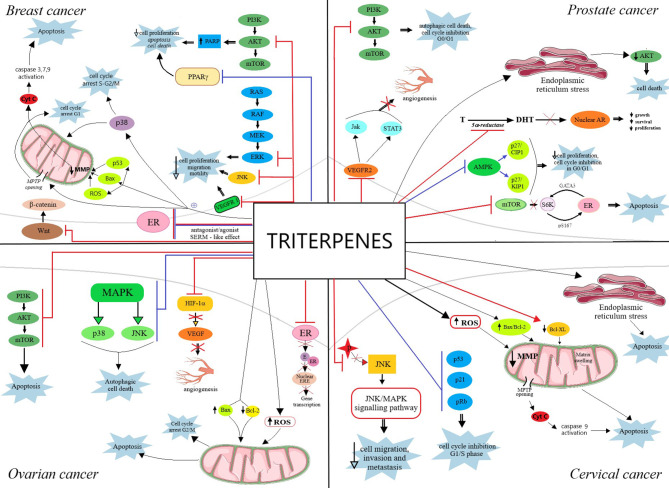
Overview of the oncogenic signaling pathways targeted by triterpenoids in reproductive hormones-dependent cancers: breast cancer, prostate cancer, ovarian cancer and cervical cancer. The red bars represent an inhibitory effect whereas blue bars represent a stimulatory effect. ER, estrogen receptor; E, estrogen; ERE, estrogen response element; AR, androgen receptor; Cyt c, cytochrome c; ROS, reactive oxygen species; T, testosterone; DHT, dihydrotestosterone. A more detailed explanation regarding the antiproliferative mechanism of action of triterpenes, in each type of cancer, is explained in detail in the main text.

An excellent review was published in 2012 by Shanmugam et al. on the topic of triterpenoids, particularly pentacyclic, and their anticancer molecular mechanisms; briefly, the authors concluded that triterpenoids are able to simultaneously target multiple oncogenic signaling pathways in numerous tumor types. In addition, the review describes several synthetic triterpenoid derivatives that were included in clinical trials; both natural and synthetic triterpenoids have the ability to act as chemopreventive and therapeutic agents in cancers sharing common molecular targets thus confirming their traditional use against inflammatory or malignant diseases (34). The oncogenic signaling pathways targeted by triterpenoids in reproductive hormones-dependent cancers are depicted in **Figure 6**.

Triterpenes exhibit strong cytotoxic effects against numerous cancer cell lines, the presence of a hydroxyl group in C28 position apparently intensifying their antitumor potency (35). The mechanisms underlying these antitumor effects are not yet completely deciphered which limits the use of triterpenes as therapeutic agents; however, most compounds exhibit apoptotic and antiangiogenic properties through the up or down regulation of various signaling pathways (36). The literature reports several cancer specific targets for triterpenes such as the proteasome, B cell lymphoma 2 (Bcl-2), NF-κB, STAT3, TNF, angiogenesis, PI3K/AKT/mTOR, and toll-like receptors (TLR) (37). An additional important limitation of triterpene use in therapy is their poor aqueous solubility; multiple attempts were conducted in order to eliminate this drawback, such as the use of hydrophilic carriers or the design of complex pharmaceutical formulations (*e.g.* nanoemulsions) (2).

The most extensively documented class of triterpenes contains the lupane-type compounds betulin, betulinic acid and lupeol; several excellent reviews have been published covering multiple aspects of their chemical and biological profiles, in particular their encouraging antitumor effects. Cháirez-Ramírez et al. described the anticancer activities of lupane-type triterpenes against breast cancer (32); molecular mechanisms have been revealed as well, mainly consisting in the modulation of proliferation or cell death-related signaling pathways (*e.g.* NF-κB, Wnt/β-catenin, PI3K/AKT, apoptosis) and resulting in extrinsic/intrinsic apoptosis and autophagy induction. The review highlighted two key aspects for the clinical use of lupane triterpenes; firstly, they exert chemopreventive effects against risk factors that might trigger malignant processes and, secondly, triterpenes act selectively on tumor cells, leaving healthy cells unharmed. In addition to their antitumor activity, triterpenes show antioxidative and anti-inflammatory properties which frequently accompany immune modulation (38–40). Due to their multiple benefits and lack of toxic side effects, vegetal triterpenes are part of health fortifying foods, natural remedies and dietary foods (38–40). Another study evaluated the main triterpenic acids such as betulinic, oleanolic and ursolic acids, which can be found in plants in unbound state; the review highlighted their antitumor effects occurred as a result of their antiproliferative, apoptotic, tumor cell cycle inhibitory and anti-angiogenic properties (31). In addition, triterpenic acids have the ability to stimulate cell differentiation and prevent tumor invasion and metastasis. An appealing application is the combination of triterpenes with low doses of consecrated clinically-effective chemotherapeutic agents, thus achieving less severe side effects and even synergistic outcomes (40).

Among triterpenes, betulin and its acid, betulinic acid, exhibit the most significant biological potential due to their large plethora of targeted pathologies such as inflammations, allergies, and most importantly, various malignancies where the two compounds were shown to inhibit tumor proliferation and angiogenesis. Betulinic acid exhibited a strong capacity to induce apoptosis in KB human cervical cancer cells through the down-regulation of specificity protein 1, highly expressed in tumors, and its downstream targets thus acting as a chemopreventive agent (41). An excellent review was published in 2019 by Hordyjewska et al. who collected all significant previously published data on betulin and betulinic acid; briefly, after a concise description of their discovery, structure and synthesis, the authors focused on a thorough presentation of their *in vitro* and *in vivo* anticancer potential, including molecular mechanisms and medical applications (42). Betulin and betulinic acid as well as some of their semisynthetic derivatives induce apoptosis through the mitochondrial pathway and can be highly effective in combination with other chemo- or radiotherapies or even with other unconventional drugs such as thalidomide; one extremely important advantage is their selectivity towards cancer cells which might improve therapeutical outcomes and decrease mortality rates.

Triterpenoid saponins exhibit a vastly diverse glycosidic structure with a plethora of biological and pharmacological properties; Koczurkiewicz et al. reviewed in 2015 the anticancer activity of saponins particularly emphasizing their anti-invasive effect. Briefly, triterpenoid saponins in low concentrations are able to inhibit cancer cell proliferation, induce apoptosis and attenuate cell invasiveness, while high concentrations produce a cytotoxic and hemolytic effect through the permeabilization of cell membranes. The authors concluded that saponins may affect the expression of oncogenes depending on the cellular context, in close relationship with the momentary genomic/proteomic cell status (43). The most frequently studied plant extracts containing triterpenoid compounds with reported *in vitro* antiproliferative activity are presented in **Table 1**.

**Table 1:** Triterpenoid compounds with reported *in vitro* antiproliferative activity.

Ring type	Basic structure	Vegetal specie	Cell line affected by its antiproliferative activity		IC_50_		Reference
	***Tetracyclic triterpenoids***						
**Cucurbitane**	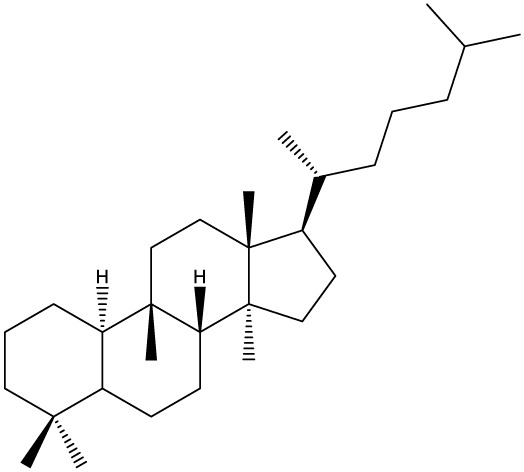	*Momordica charantia*	Human breast carcinoma MDA-MB-231 and MCF-7 cell line	14.3 and 17.6 μM			(44–48)
			Human breast carcinoma MCF-7 cell line and Du145 prostatic carcinoma cell line	41.74 and 30.56 μmol/L; 89.72 and 61.36 μmol/L			
			Human breast adenocarcinoma SK-BR-3 Cell Line	6.6 μM			
		*Hemsleya penxianensis*	Breast carcinoma MCF-7 cell line and Human cervical carcinoma HeLa cell line	2.25 to 49.44 µM			
		*Hemsleya pengxianensis* var. *jinfushanensis*	Prostate carcinoma Du145 cell line				
**Cycloartane**	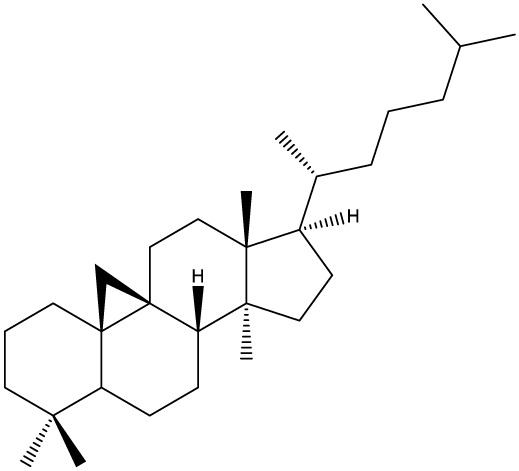	*Cimicifuga foetida*	Human breast carcinoma MDA-MB-231 cell line	6.74 μM and 10.21 μM			(49–51)
			Breast carcinoma MCF-7 cell line	5.86 - 14.21 μm			
		*Cimicifuga yunnanensis*	Human breast cancer cells, MCF-7, MDA-MB-231 and SK-BR3	0.1 – 5.5 µg/ml			
					
**Dammarane**	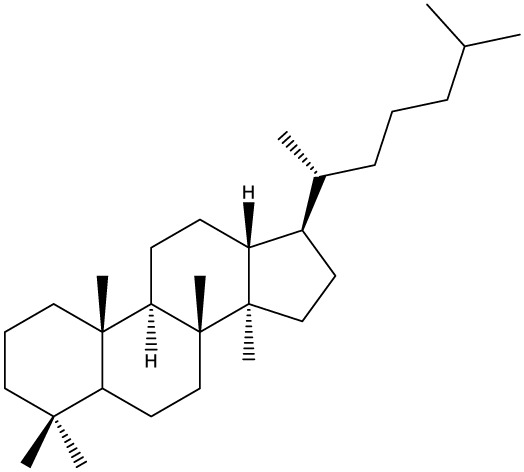	*Gynostemma pentaphyllum*	Breast carcinoma MCF-7 cell line and human breast surface epithelial cells MCF-10a	6.85–52.4 μM			(52–54)
		*Panax ginseng*	Human breast cancer cell line MCF-7 and prostate carcinoma PC-3 cell line	10–60 μM			
		*Cleome khorassanica Bunge & Bien*	Prostate cancer cell lines DU-145 and LNCaP	11.07–54.82 μM			
					
**Euphane**	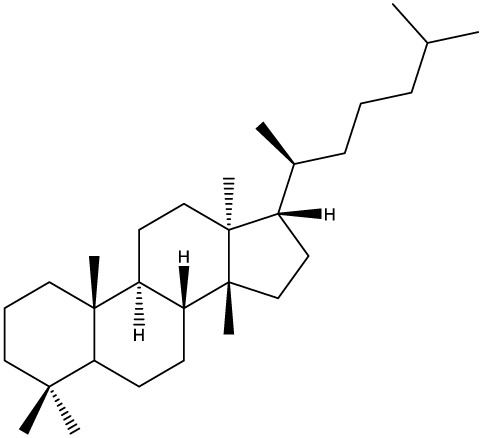	*Cassipourea lanceolata* *Hibiscus tiliaceus* *Phomopsis chimonanthi*	Human ovarian cancer A2780 cell line Human cervical carcinoma HeLa cell line Human breast carcinoma MDA-MB-231 cell line	17 μg/ml 11.5 μmol/L 19.87 μM			(55–57)
					
Lanostane	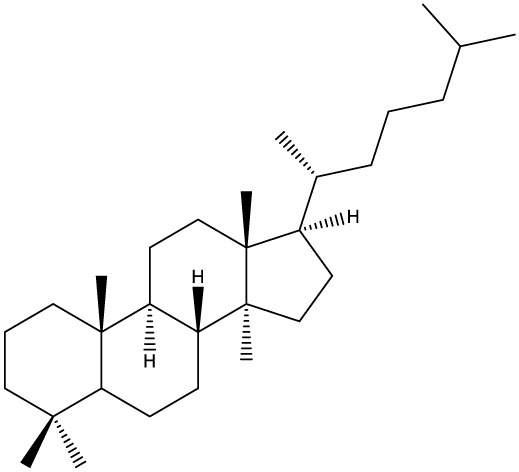	*Ganoderma luteomarginatum*	Human cervical carcinoma HeLa cell line	1.29 μM			(58, 59)
		*Ganoderma lucidum*	Human breast carcinoma MDA-MB-231 cell line	21.2 - 158.7 μM			
					
					
**Prostane**	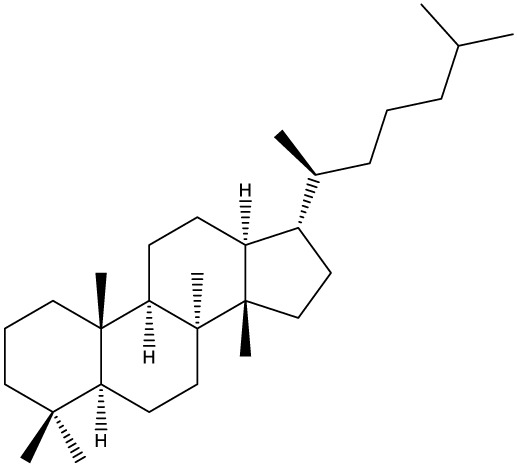	*Alisma plantago-aquatica L*. var. *orientale Samuelson*	Human ovary adenocarcinoma SK-OV3 cell line	7.5 µg/ml			(60)
					
**Tirucallane/Apotirucallane**	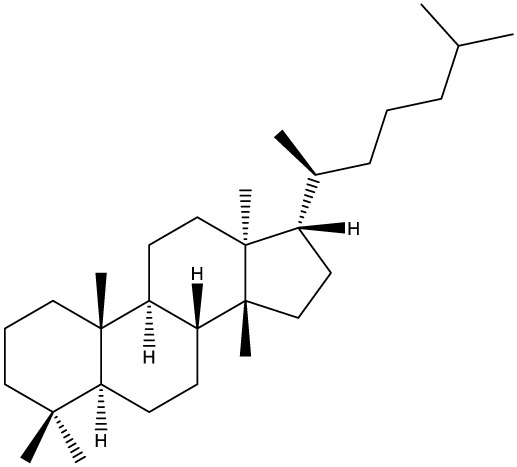	*Scorzonera divaricate*	Human cervical carcinoma HeLa cell line	24.5 μM			(55, 61–66)
		*Hibiscus tiliaceus*	Human cervical carcinoma HeLa cell line	19.9 μmol/L			
		*Amphipterygium adstringens*	Breast carcinoma MCF-7 cell line and prostate carcinoma PC-3 cell line	18.4–31.5 μM and 27.4–32.6 μM			
		*Luvunga scandens*	Breast carcinoma MCF-7 cell line	13.8 μM and 27.5 μM			
		*Walsura chrysogyne*	Breast carcinoma MCF-7 cell line	32.2 μM and 62.5 μM			
		*Ficus carica*	Breast carcinoma MCF-7 cell line	17.94 μM			
		*Picrasma quassioides*	Breast carcinoma MCF-7 cell line	8.5–10 μM			
					
					
	***Pentacyclic triterpenoids***						
	**Baccharene type**						
**Friedelane**	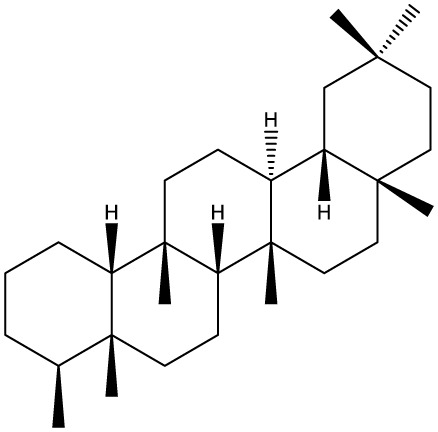	*Siphonodon celastrineus Griff*	Human cervical carcinoma HeLa cell line and human breast carcinoma MDA-MB-231 cell line	Not cytotoxic			(67–70)
		*Anchietea pyrifolia*	Human cervical carcinoma HeLa cell line	5.7–45.0 μM			
		*Maytenus robusta*	Murine 4T1 breast cancer cell	9.80 µM, 1.23 µM, 98.10 µM, 58.10 µM, 23.10 µM, 11.00 µM			
		*Garcinia celebica*	Human breast cancer MCF-7 cell line	82 and 70 µM			
				
**Glutinane**	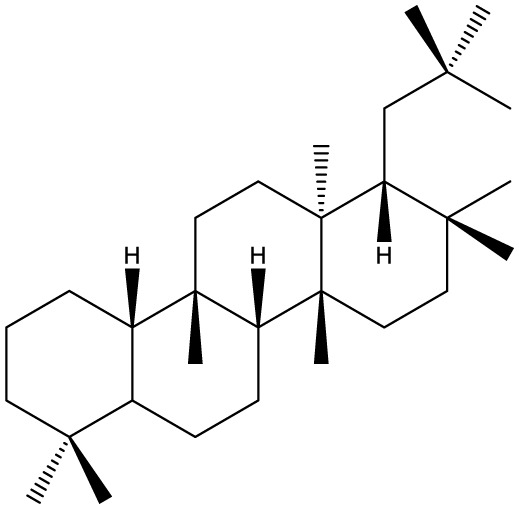	*Walsura pinnata*	Prostate adenocarcinoma PC-3 cell line Breast adenocarcinoma (MDA-MB-231)	55.3 ± 3.9 µM			(71, 72)
			Breast adenocarcinoma MCF-7 cell line Cervical adenocarcinoma (HeLa S3)	92.9 ± 3.1 µM			
			Cervical carcinoma (SiHa)	60.9 ± 2.3 µM			
			Prostate carcinoma (DU 145)	59.7 ± 0.9 µM >100^b^			
		*Tibouchina urvilleana*	Prostate carcinoma PC-3 cell line Breast carcinoma MCF-7 cell line	87.2 ± 2.1 µM			
					
**Lupane**	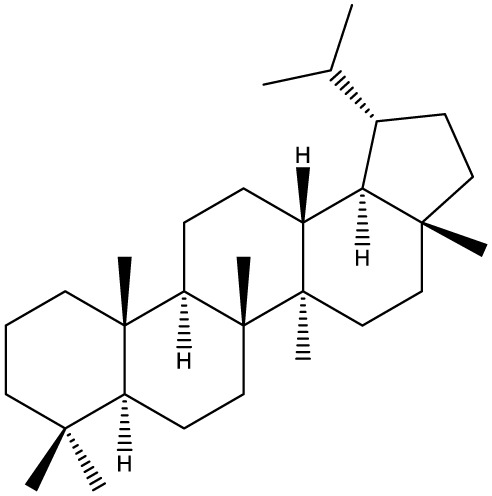	*Siphonodon celastrineus Griff*	Human cervical carcinoma HeLa cell line and human breast carcinoma MDA-MB-231 cell line	Not cytotoxic			(68, 73–75)
		*Davidia involucrata*	Breast carcinoma MCF-7 cell line	7.26–12.37 μM			
		*Akebia* *Trifoliate*	Human cervical carcinoma HeLa cell line	81.49 μM			
		*Potentilla* *discolor Bunge*	Breast carcinoma MCF-7 cell line	30.78 μM			
**Multiflorane**	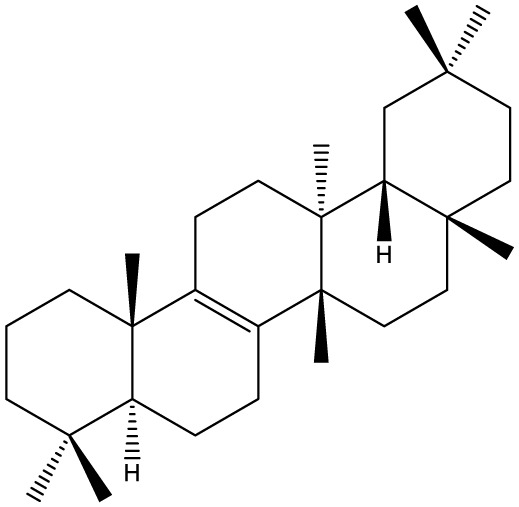	*Trichosanthes kirilowii*	Human ovarian carcinoma OVCAR-3 and OVCAR-6 cell line	1.79 and 2.06 μM			(74, 76, 77)
		*Akebia trifoliate*	Human cervical carcinoma	26.5–51.9 μM			
		*Turraea* spp.	HeLa cell line A2780 human ovarian cancer cell line	20 μM			
**Oleanane**	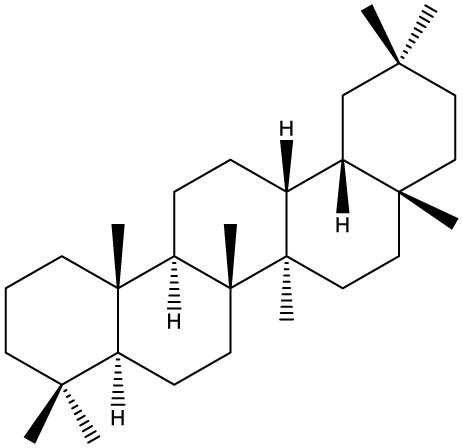	*Siphonodon celastrineus Griff*	Human cervical carcinoma HeLa cell line and human breast carcinoma MDA-MB-231 cell line	Not cytotoxic			(68, 72, 78–80)
		*Luffa cylindrical*	Human breast cancer MDA-MB-231 and MCF-7 cell line	41.72 μM and 48.17 μM			
		*Stauntonia brachyanthera*	Human breast cancer MDA-MB-231cell line	27.73 μM, 97.33 μM and 29.23 μM			
		*Tibouchina* *Urvilleana*	Breast carcinoma MCF-7 cell line				
		*Clematis* *ganpiniana*	Invasive ductal carcinoma BT474 and SUM1315 cell lines and human breast adenocarcinoma cell lines MCF-7, MDA-MB-231	5, 10, 20, 40 and 80 mg/ml			
**Taraxerane**	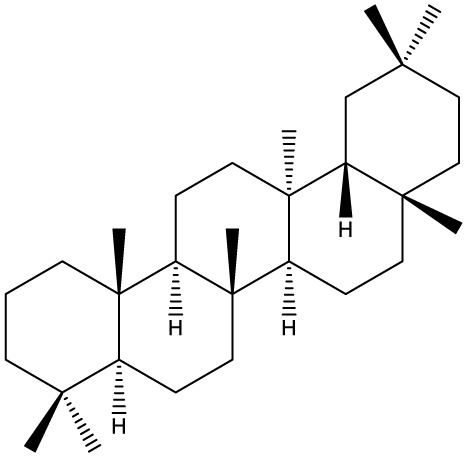	*Saussurea graminea*	Human cervical carcinoma HeLa cell line and breast carcinoma MCF-7 cell line	9.47 -30.14 μM and 9.27 -30.35 μM			(72, 73, 81)
		*Davidia involucrate*	Breast carcinoma MCF-7 cell line	41.34 μM and 42.54 μM			
		*Tibouchina* *urvilleana*	Breast carcinoma MCF-7 cell line				
**Ursane**	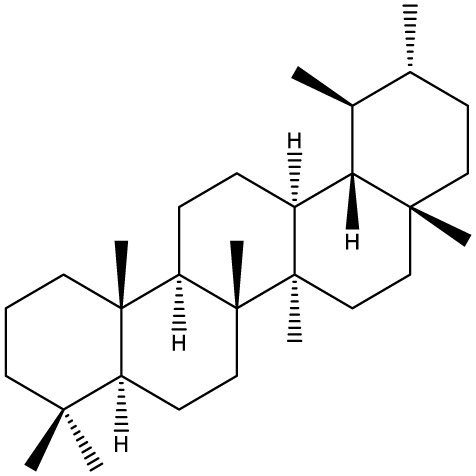	*Siphonodon celastrineus Grif*	Human cervical carcinoma HeLa cell line and human breast carcinoma MDA-MB-231 cell line	Not cytotoxic to any of the cell lines tested			(68, 72, 73, 82)
		*Davidia involucrate*	Human breast cancer MCF-7 cell line				
		*Crataegus pinnatifida*	Human ovary adenocarcinoma SK-OV3 cell line	36.3–76.4 μM			
		*Tibouchina* *urvilleana*	Human breast cancer MCF-7 cell line	3.27 μg/ml			
	**Hopane type**						
**Gammacerane**	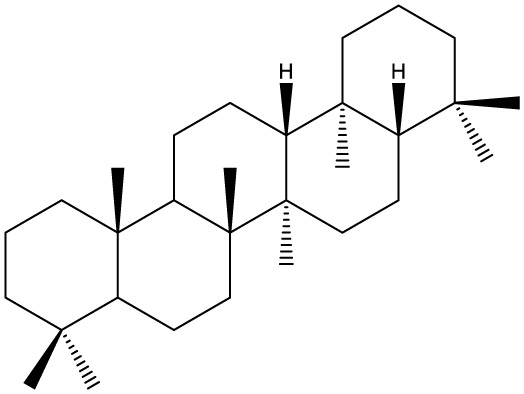	*Abies veitchii A. mariesii*	Human endometrial carcinoma RL-95 cell line				(83)
					
**Hopane**	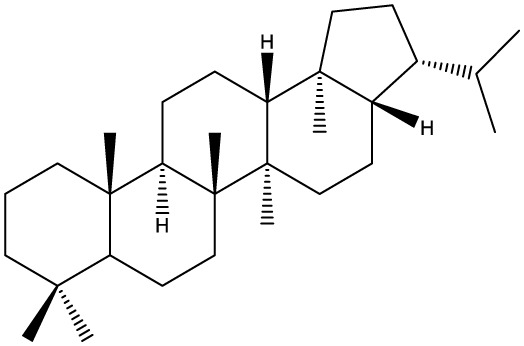	*Parmotrema sancti-angelii*	Human breast cancer MCF-7 cell lin		47.08 ± 2.64 µg/ml		(84–86)
		*Cnidoscolus spinosus*	Human breast cancer MCF-7 cell line and prostate carcinoma PC-3 cell line		Not cytotoxic		
		*Megacodon stylophorus*	Human cervical carcinoma HeLa cell line and breast cancer MCF-7 cell line		3.6 µM; 7.5 µM		
					
**Neohopane**	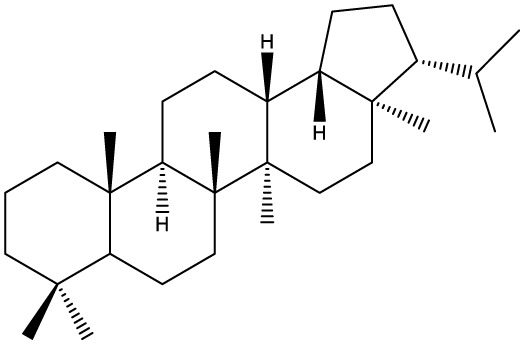	*Carissa carandas L*.	Human cervical carcinoma HeLa cell line and prostate carcinoma PC-3 cell line			6.87 µM; 12.60 µM	(87)
					

Nonetheless, triterpenes have produced rather poor *in vivo* results (**Table 2**) (88, 107), despite the promising *in vitro* data, presumably due to their almost complete insolubility in water; furthermore, the *in vitro* experiments may be biased by the unavoidable use of organic solvents such as DMSO. Therefore, many studies have focused on finding solutions to preserve the pharmacologically useful properties of triterpenes which make them candidates as lead compounds while eliminating their damaging physicochemical flaws. Such an approach was the development of semisynthetic derivatives, some of which currently undergoing clinical trials (108). Among the huge number of semisynthetic compounds reported in the literature all around the world, oleanane-type triterpenoids such as 2-cyano-3,12-dioxooleana-1,9(11)-dien-28-oic acid (CDDO) and its methyl ester (CDDO-Me) and imidazolide derivatives obtained through modifications of the C17 position have been particularly noted as anticancer agents. CDDOs have been revealed to inhibit tumor growth and to induce apoptosis through their oxidative stress response effects and also to suppress the production of nitrogen oxide and various enzymes involved in the carcinogenesis process (109, 110). CDDO-Me has proven its efficiency in hormone-dependent cancers, such as ovarian (111) and breast (112) cancers, but also in a wide range of other types of tumors (113, 114). Therefore, it was subjected to clinical trials under the name bardoxolone methyl (108) and is currently waiting for FDA approval to be used as treatment of advanced chronic kidney disease caused by Alport syndrome. An analogue of CDDO, cyano enone of methyl boswellate (CEMB), was synthesized by Ravanan et al. in 2011 who conducted biological tests on several cancer cell lines including prostate cancer; the synthetic triterpenoid acted as a cytotoxic agent in a dose-dependent manner with submicromolar LD50 values by activating initiator and effector caspases involved in extrinsic and intrinsic apoptosis pathways (115).

**Table 2:** Triterpenoid compounds with reported *in vivo* antiproliferative activity.

Ring type	Compound name	In vivo cancer models	Effects and Mechanisms	Dose; duration; route	Reference
	***Tetracyclic triterpenoids***					
**Cycloartane**	*23-epi-26-deoxyactein*	Breast cancer (MDA-NB-231 cells) liver metastasis nude mice model	Tumor growth inhibition	2 mg/kg; 2 weeks	(51)	
**Cucurbitane**	*Cucurbitane B* *Cucurbitane B* *Cucurbitane B* *Cucurbitane B* *Cucurbitane D*	MDA-MB-468 cells implanted into the breasts of nude mice MDA-MB-231 human breast cancer orthotopic xenografts in immunodeficient Sprague-Dawley mice PC-3 and LNCaP xenograft male athymic mouse model MDA-MB-231 cells and 4T-1 cells orthotopically implanted in the mammary fat pad of female athymic BALB/c mice and nude mice Athymic nude mice with cervical cancer (CaSki) cell-derived orthotopic xenograft tumors	Tumor volume was reduced by 55% Significantly reduced tumor volume Significantly reduced the rate of *in vivo* tumor-formation Tumor size reduction by 79% (vs. control) at day 31 Tumor volume was reduced by 55% (MDA-MB-231 cells) and 39% (4T-1) vs. control Significant reduction of tumor volume and weight; decreased expression of pAKT and pSTAT3 proteins	1.0 mg/kg; 6 weeks; i.p 0.5 mg/kg and 1.0 mg/kg; 3 times/week; 36 days 0.1 µmol, 5 days, oral gavage; 2 weeks pre-treatment before cancer cell implantation; 2 mg/kg daily; 60 days; oral gavage. 1 mg/kg body weight; intra-tumoral	(88–93)	
**Tirucallane**	*Tirucallic acid*	Prostate Cancer (PC-3) Xenografts in male NMRI/nu-nu mice	Decreased the growth of pre-established prostate tumors; inhibited AKT phosphorylation and down-regulated the nuclear localization of the AKT-dependent nuclear phosphoprotein c-Myc.	10 µmol/kg daily; 2 weeks; i.p	(94)	
	***Pentacyclic triterpenoids***					
**Friedooleanane**	*Pristimerin* *Pristimerin*	BALB/c nude mice xenografted with MDA-MB-231 cells in mammary fat pads NOD.CB17-Prkdcscid/J mice xenografted with human breast adenocarcinoma MCF-7 and MDA-MB-231 cells	Inhibition of tumor growth and invasiveness Delayed tumor growth and decreased tumor size; reduced the expression of AKT1, increased the negative regulator PTEN, the cleavage of PARP and levels of active caspase 3 and/or 7	1 mg/kg body weight every other day; 12 days 12 mg/kg; 2 weeks; i.p	(95, 96)	
**Lupane**	*Betulinic acid* *Betulinic acid* *Lupeol* *Betulinic acid*	Female athymic BALB/c nude mice with xenografted MDA-MB-231 cells Male athymic BALB/c nude mice bearing LNCaP cell (androgen-sensitive human prostate adenocarcinoma cells) xenografts Athymic mice bearing C4-2b and LNCaP cell–originated tumor Zebrafish breast cancer xenotransplantation model and transgenic MMTV-PyVT+/− breast cancer spontaneous mouse model	Tumor growth inhibition; miR-27a suppression and induced expression of the miR-27a-regulated gene “ZBTB10” Tumor growth inhibition; Decreased the expression of VEGF and induced proteasome-dependent degradation of the transcription factors specificity protein 1 (Sp1), Sp3, and Sp4 Decreased tumor volume Inhibition of breast cancer growth Inhibition of glycolytic activity	20 mg/kg/day- every second day; 25 days 10 and 20 mg/kg/day – every second day for 14 days 40 mg/kg; 3 times/week; 12 weeks; i.p 250 mg/kg; i.p	(16, 97–100)	
**Oleanane**	*AMR-Me (methyl-25-hydroxy-3-oxoolean-12-en-28-oate)* *Oleanolic acid* *Oleanolic acid*	DMBA-induced mammary tumorigenesis in female Sprague–Dawley rats BALB/c nude male mice PC-3 cells xenograft model Nude mice HeLa cells xenograft model	Inhibited tumor incidence and burden, reversed histopathological alteration without toxicity, suppressed abnormal cell proliferation; upregulation of pro-apoptotic protein Bax and down-regulation of antiapoptotic Bcl2. Tumor growth suppression; induction of apoptosis and G0/G1 phase cell cycle arrest *via* the regulation of PI3K/AKT pathway. Significant reduction of tumor mass and size	0.8, 1.2, and 1.6 mg/kg; 3 times/week; 18 weeks; p.o 150 mg/kg/day; 21 days; i.p 40 mg/kg and 80 mg/kg; 15 days; i.p	(101–103)	
**Ursane**	*Ursolic acid* *Ursolic acid* *Ursolic acid*	Ovariectomized C57BL/6 mice xenografted with MMTV-Wnt-1 mammary tumor cells DMBA-induced mammary tumorigenesis in female Sprague–Dawley rats LNCaP prostate tumor xenografts in athymic nude mice	Decreased tumor cell proliferation Failed to suppress DMBA-induced mammary tumors Tumor growth inhibition; induction of apoptosis, decreased expression of p-AKT and p-mTOR	54, 106, or 266 mg/kg body weight/day; 3 weeks; diet 200 mg/kg/day; 5 days; i.p 20 mg/kg and 40 mg/kg; 5 days/week, 3 weeks; i.p	(104–106)	

Pentacyclic triterpenes are molecules with high lipophilicity and implicitly, very low hydrosolubility, an essential aspect that influences their bioavailability after oral administration. These natural molecules violate several criteria of the “Lipinski’s rule of five” such as, the octanol/water partition coefficient (for the vast majority ranging between 6 and 8) or the number of H-bond donors/acceptors (116). These are important factors that determine both the ability of a certain molecule to cross biological membranes and the physical interaction of the substance with the aqueous biological environment (117, 118). Although natural sources of pentacyclic triterpenes provide a quantifiable intake of triterpenes into the bloodstream, they are contained in complex “natural formulations” that can drastically influence bioavailability; in addition, natural sources may contain glycosylated derivatives or esters with superior pharmacological properties (119). However, even if *in vitro* bioavailability studies performed on pure substances show promising results, this data is not usually extrapolable *in vivo*, where pure substances end up exhibiting very low bioavailability values (119). The pentacyclic triterpenes’ poor aqueous solubility and low bioavailability hinder their potential to generate new anticancer therapies. The field of nanotechnology has provided new options to enhance the solubility, stability, bioavailability, and delivery of pentacyclic triterpenes, thus optimizing their clinical efficacy (120); liposomes, nanoemulsions, various nanoparticles (i.e. solid lipid NP, polymeric NP, magnetic NP, nanocapsules, nanospheres, dendrimers, etc.), cyclodextrin complexes and other nanosystems with ursolic, oleanolic and betulinic acid were designed, prepared and thoroughly characterized revealing high anticancer efficiency and selectivity doubled by low toxicity.

## Triterpenes as Therapeutic Agents in Sex Hormone-Dependent Cancers

As previously stated, triterpenes can be found as secondary metabolites in a large variety of vegetal species; they have been investigated in terms of biological effects either as crude/enriched extracts or as isolated compounds. The literature reports comparative studies between whole extracts and isolated compounds, with pros and cons for each of them, for the prevention and treatment of numerous diseases. While isolated compounds may be chosen for their high biological activity they can also show disadvantages such as weaker effects than the whole extract and unaffordable prices (121); such weaker activity may be explained by a synergistic effect of combined compounds in plant extracts as well as the presence of substances which inhibit multi-drug resistance. Triterpenes isolated from *Ganoderma lucidum* (Leyss.:Fr.) Karst were shown to reveal antitumor, anti-inflammatory and immunomodulatory activities; their anticancer effect is a result of combined cytotoxic, cytostatic and anti-metastasis properties manifested *in vitro* and *in vivo* in numerous cancer types including reproductive hormone-dependent cancers (122, 123). A key observation emphasized by Cheng et al. is the fact that future clinical trials should focus on chemically characterized vegetal extracts in order to develop an anticancer activity-associated chemical fingerprint which might orientate ulterior studies.

This review includes studies of both crude and triterpene-enriched extracts as well as isolated triterpenoids as therapeutic agents in reproductive hormones-dependent cancers.

### Breast Cancer

An excellent review has been published by Bishayee et al. on the topic of triterpene use against breast cancer; briefly, the authors reviewed the main types of triterpenoids tested *in vitro* on various breast cancer cell lines with some of these classes (cucurbitanes, friedelanes, oleananes, ursanes) being also subjected to *in vivo* assessments (124). The authors emphasized the unique anticancer mechanisms induced by triterpenes which may be synergistically expressed when combinations of phytochemicals are used; also, triterpenes are able to enhance the therapeutic efficacy of conventional chemo- or radiotherapies while limiting their side effects. The clinical use of triterpenes might benefit of their chemical derivatization to more water soluble compounds or their formulation into nanoparticles with improved pharmacokinetic profile (124).

The avocado plant (*Persea americana* Mill.) is widely used around the world as food or in traditional medicine; its seed, usually wasted, contains a mixture of compounds, including triterpenoids (125). A recent study showed that both the crude extract and the isolated compounds act as antiproliferative agents on several tumor cell lines; the IC_50_ values below 100 µg/ml displayed by the whole or fractionated extracts indicate their potential in chemoprevention while the IC_50_ value of 62 mg/ml of the isolated compound indicates a significant cytotoxic effect against MCF-7 breast cancer cells (125).

Triterpene glycosides extracted from the black cohosh (*Actaea* syn. *Cimicifuga racemosa* L. NUTT) were investigated in terms of antiproliferative agents and apoptosis inducers on ER+ MCF-7 breast cancer cells (126); the whole extract exhibited a dose-dependent down regulation of cell proliferation with a comparable degree of apoptotic MCF-7 cells. Interestingly, the triterpenoid fraction induced apoptosis at much lower concentrations than needed for cell growth inhibition; additionally, cell death required a metabolic activation system. The investigation of the underlying mechanism of apoptotic cell death induced by triterpenes revealed useful data for future therapeutic alternatives; thus, 3β,7β-dihydroxy-25-methoxycucurbita-5,23-diene-19-al isolated from wild bitter gourd (*Momordica charantia* L.) activates the peroxisome proliferator-activated receptor (PPARγ) and subsequently modulates multiple PPARγ-targeted signaling pathways involving numerous effectors such as cyclin D1, CDK6, Bcl-2, XIAP, cyclooxygenase-2, NF-κB, ERα, AKT, and AMPK (44) finally resulting in cell apoptosis and autophagy. The selective activity of *Momordica charantia* L. triterpene-rich extracts on ER positive cell lines was found to be correlated with that of selective estrogen receptor modulators (SERM). The study of Hsu et al. demonstrated the agonistic/antagonistic effect of a cucurbitane-type triterpenoid form *Momordica charantia* L. on ERα and ERβ (127). Actein, a cycloartane triterpene glycoside extracted from the rhizomes of *Cimicifuga foetida* L. was documented as a selective cell growth inhibitor of breast cancer cells as well as able to act in a synergic manner with consecrated chemotherapy agents; Yue et al. investigated in 2016 the activity of actein on tumor angiogenesis, an essential step in tumor development and metastasis; *in vitro* results showed that actein suppressed the expression of VEGFR1, pJNK, and pERK subsequently inhibiting cell proliferation, migration and motility. When tested *in vivo* actein significantly inhibited blood vessel formation and decreased tumor size and metastasis through reducing the expression of angiogenic proteins (CD34 and Factor VIII) and metastasis-related VEGFR1 and C-X-C chemokine receptor type 4 (CXCR4) genes (128). The anticancer activity of triterpene glycosides was also documented by molecular docking conducted on phytochemical compounds from *Begon*ia sp., thus encouraging their direct isolation and assessment (129). The antiproliferative action of cycloartane-type triterpenes in sex-hormone dependent cancer can be corelated with the antagonist effect towards ER. Studies have showed that cycloartane triterpenoids, isolated from *Schisandra glaucescens* Diels, present an antagonistic activity against ERα and ERβ with EC50 values of 2.55 and 4.68 µm (130). This study also showed that the potency of the tested compound is equivalent to that of tamoxifen (130).

Saikosaponins are triterpene saponin glycosides (131) extracted from the roots of *Bupleurum chinense* DC. which was used extensively in Traditional Chinese Medicine as adjuvant treatment for breast cancer; network pharmacology and bioinformatics analysis was employed to unravel the biological mechanisms and therapeutic targets of these compounds (132). The study revealed that saikosaponins are able to target a multitude of proteins such as the apoptosis regulator Bcl-2, CXCR4, ATP-dependent RNA helicase DDX5, protein kinase C alpha and proto-oncogene tyrosine-protein kinase Src; in addition, in terms of involved mechanisms against breast cancer, saikosaponins regulate several key signaling pathways (*e.g.* PI3K-AKT signaling pathway, EGFR tyrosine kinase inhibitor resistance, etc.) (132). Seven triterpenoid saponins were extracted from *Gleditsia sinensis* Lam. and investigated in terms of chemical structure and biological activity; a significant cytotoxic effect was noticed against breast cancer MCF-7 cells by induced apoptosis (133). The authors concluded that an additional hydroxyl group in C16α position favors the compound’s cytotoxic activity.

Marine triterpene glycosides extracted from various species of sea cucumbers exhibit anticancer effects on numerous cancer cell lines including breast cancer (134); their molecular mechanisms can be understood in-depth by thoroughly investigating their structure-activity relationship. Studies in this regard showed: 1) the importance of the mutual positions of keto groups and double bonds in the aglycone structure; 2) the necessity of a linear tetrasaccharide chain for a membranolytic activity; 3) the presence or absence of a sulfate group in the sugar chain; structural differences trigger different biological activities. As a result, several mechanisms have been identified: inhibition of proliferation, apoptosis induction, cell cycle arrest, antimetastatic activity (134).

One of the most active anticancer triterpenes is betulinic acid which was extensively studied and showed strong selective cytotoxic effects on numerous cancer cells; its derivatives exhibit the same useful anticancer properties but improved pharmacokinetic profile. Betulinic acid and its derivatives are highly active on a multitude of cancer cell lines including sex hormone-dependent cancers (135). Moreover, betulinic acid displays protective and therapeutic efficiency on cancer-associated bone pathologies such as breast cancer metastasis (136); its effects in the cycle of osteolytic bone metastasis were investigated, revealing that the cell viability and synthesis of the parathyroid hormone-related protein (PTHrP) which acts as a major osteolytic factor were significantly reduced. In addition, betulinic acid caused the downregulation of specific proteins thus inhibiting and preventing bone loss in patients with bone metastasis and therapeutically-induced estrogen deficiency. Betulinic acid’alcohol, betulin, was extracted together with several other triterpenoids, including betulinic acid, from the Chinese herb *Hibiscus syriacus* L.; when tested on breast cancer cells they showed inhibitory effects against cell proliferation and migration while normal mammalian epithelial cells lacked inhibitory phenomena (137). In terms of underlying mechanisms, betulin derivatives induced apoptosis and cell cycle arrest by activating p21 expression.

A thorough investigation of the anticancer and chemopreventive mechanism was conducted on asiatic acid (2,3,23-trihydroxyurs-12-en-28-oic acid), a triterpenoid isolated from *Centella asiatica* (L.) Urb. and found strongly active as cell proliferation inhibitor in both ER positive (MCF-7) and negative (MDA-MB-231) breast cancer through the activation of p38 and ERK1/2 kinases pathways (138); the investigators established that asiatic acid induce mitochondrial apoptosis and S-G2/M cell cycle arrest by modulating the ERK1/2 cascade and the p38 pathway, respectively. Cell cycle arrest was indicated by the increased levels of p21/WAF1 and reduced concentrations of cyclinB1, cyclinA, Cdc2 and Cdc25C without involving the p53 pathway; also, Cdc2 function was reduced through increased levels of the p21/WAF1/Cdc2 complex and inactivated phospho-Cdc2 and phospho-Cdc25C.

A friedolanostane triterpenoid (methyl−3α,23−dihydroxy−17,14−friedolanstan−8,14,24−trien−26−oat) was isolated from *Garcinia celebica* L. and tested on MCF-7 breast cancer cell; the compound inhibited cell proliferation in a time- and dose-dependent manner by inhibiting the oncogenic protein AKT and increasing the expression of the poly (ADP-ribose) polymerase (PARP) protein thus promoting apoptosis and cell death (67). A natural friedo-oleanane triterpene, celastrol, found in the *Celastraceae* family, was the topic of a comprehensive review published in 2018 by Yadav et al. who emphasized its activity on multiple proinflammatory, angiogenic and metastatic proteins thus inhibiting cancer growth, survival and metastasis; due to its large plethora of biological effects exerted through the modulation of a variety of intra- and extracellular signaling pathways, celastrol was the subject of numerous patents published between 2011 and 2017 and was embedded in modern complex nanoformulations (139).

An oleanane type triterpenoid saponin, tubeimoside-1 (olean-12-en-28-oic acid), extracted from a traditional Chinese medicinal plant, *Bolbostemma paniculatum* (Maxim.) Franquet, exhibit various pharmacological activities, including antitumor against a wide range of cancer types; its antitumor effect on breast, prostate, cervical and ovarian cancer has been documented (140). Peng et al. conducted in 2016 a study focused on the underlying molecular mechanisms of action of tubeimoside-1 on human breast cancer (141). Despite previous research claiming the antiproliferative and pro-apoptotic effects on several cancer cell lines through the activation of caspase-dependent pathway, inhibition of Wnt/β-catenin signaling pathway and induction of a p53/MDM2-dependent mechanism, the study revealed a weak influence of tubeimoside-1 on the cellular viability and proliferation of breast cancer cells. However, at the same concentration, tubeimoside-1 attenuated CXCR4 expression in breast cancer, thus inhibiting CXCL12-induced cell invasion and metastasis; the anti-metastasis effect might offer cancer patients better chances at long-term survival (141). Oleanane triterpenoids and their potential to treat breast cancer were the subject of a review published in 2014 by Parikh et al.; briefly, natural and semisynthetic oleananes display strong antiproliferative and anti-metastasis effects on a large range of breast cancer cell lines, mainly through apoptosis and cell cycle arrest (112). The authors concluded that a combination of phytochemicals, including oleanane derivatives, might serve as multitargeted therapy and prevention in breast cancer but can be also administered in association with synthetic anticancer drugs, in order to improve their efficacy and reduce their toxic side effects; additionally, complex pharmaceutical formulations (*e.g.* nanoparticles, liposomes, colloids, etc.) might be needed in order to overcome oleanane poor bioavailability.

A novel oleanane-type triterpene saponin D rhamnose β-hederin was isolated from *Clematis ganpiniana* and revealed a strong antiproliferative and apoptotic activity on various breast cancer cell lines through inhibiting the PI3K/AKT and activating the ERK signaling pathways; additionally, the compound regulated the ratio of proapoptotic and antiapoptotic Bcl-2 proteins and induced mitochondrial membrane depolarization, thus releasing Apaf-1 and cytochrome C into the cytosol where they activated caspases 9 and 3 (78).

Pro-apoptotic effects resulting in cell death were described for semisynthetic pentacyclic triterpenes through mitochondrial depolarization in breast cancer cell lines; additional research highlighted the loss of the mitochondrial membrane potential (MMP) causing inhibition of cell proliferation and cell cycle arrest in the G1 phase due to the strong induction of permeability transition pore at mitochondrial (MPTP) level (142). The semisynthetic compounds were lupane-type derivatives designed to increase the aqueous solubility of betulin and betulinic acid based on the assumption that it hampers their clinical use; their mechanism of mitochondrial perturbation is similar to the one of betulinic acid which triggers cancer cells apoptosis through the permeabilization of the mitochondrial outer membrane (142). Five cycloartane triterpenoids were also identified to act at mitochondrial level when tested on the breast cancer cell line MCF-7 and its corresponding drug resistant subline R-MCF-7 (143); the five compounds isolated from *Cimicifuga yunnanensis* P.K.Hsiao stimulated the expression of p53 and Bax, resulting in the loss of mitochondrial potential and, subsequently, the activation of caspase-7, followed by apoptosis and cell death.

The active hexane and chloroform extracts, respectively, of the mangrove plant (*Scyphiphora hydrophyllace*a C.F. Gaertn) were used as source of isolated compounds—ursolic, oleanolic, and eichlerianic acid—which were subsequently tested as cytotoxic agents against the ER positive (MCF-7) breast cancer cells; all three compounds showed significant growth inhibition effects accompanied by cell morphological changes (144). Ursolic acid isolated from the ethyl acetate fraction of *Betula utilis* D.Don methanolic extract was found to be highly effective as cytotoxic agent on MCF-7 breast cancer cells while non-tumorigenic breast epithelial MCF-10A cells remained unaffected, thus emphasizing a selective anticancer activity; an apoptotic effect was identified and attributed to the up-regulation of DR4, DR5 and PARP cleavage (145). In addition, ursolic acid induced the generation of intracellular ROS and the disruption of the mitochondrial membrane potential while also inhibiting cell migration, thus showing potent anticancer and anti-metastasis activity. One new ursane derivative, 2β,3β,19α-trihydroxyurs-12-en-28-oic acid, isolated from air-dried stem bark of *Vitellaria paradoxa* C.F.Gaertn. together with several known triterpenes and identified as cytotoxic on breast cancer cells allowed the investigation of structure-activity relationships; methylation of compounds led to the synthesis of methyl esters which showed improved anticancer activity compared to the parent molecules presumably due to their decreased polarity and lack of charge which favored their passive cell membrane permeability (146).

Two lupane-type triterpenic acids, 2α-hydroxy-3-oxo-lup-12(13),20(29)-dien-27,28-dioic acid and 2α, 27-dihydroxy-3-oxo-lup-12(13),20(29)-dien-28-oic acid, were isolated from the apple peels (*Malus domestica* Borkh.) by methanolic extraction. The two compounds were chemically characterized and biologically tested as antiproliferative agents, revealing strong cytotoxic activity against the CRL-2351 breast cancer cell line (147). Most important, the structure-activity relationship study conducted by the same authors highlighted the importance of the carboxyle group for the cytotoxic activity, which acts as proton acceptor; in addition, the antiproliferative activity seems to be directly dependent on the number of hydroxyl groups which act as proton donors. Another 13 compounds were extracted from the peels of *Malus pumila* Mill. and revealed as potent antiproliferative agents against MCF-7 human breast cancer cells in a dose-dependent manner (148).

### Ovarian Cancer

The echinocystic acid and its glycosides bearing one or two glucose moieties isolated from *Eclipta prostrata* (L.) L., already known for several biological effects, were found active as cytotoxic agents against ovarian and endometrial cancer. Interestingly, eclalbasaponin II which possess one glucose moiety, exhibited a much stronger cytotoxic activity than its aglycone, echinocystic acid, and the other glycoside, eclalbasaponin I, with two glucose moieties (149). Eclalbasaponin II has the ability to arrest cell cycle in the G1 phase, induced apoptosis and cell death through autophagy by regulating the JNK, p38, and mTOR signaling pathways in SKOV3 and A2780 human ovarian cancer cells.

Ursolic acid, a pentacyclic triterpene with documented activity against breast and cervical cancer, was also subjected to biological testing in order to assess its potential to exert cytotoxic effects on ovarian cancer cells (150). A significant dose-dependent reduction in the SKOV-3 ovarian carcinoma cell viability was recorded accompanied by much lower effects on normal ovarian surface epithelial cells thus emphasizing a selective anticancer activity. The study of the underlying mechanisms revealed an induction of apoptotic cell death due to increased Bax and decreased Bcl-2 levels, a dose-dependent cell cycle arrest in the G2/M phase and an increased ROS production; the inhibition of the I3K/AKT signaling pathway was recorded. Ursolic acid was also reported as a moderate antiestrogenic compound. In silico and biological evaluations showed that at high doses, the pentacyclic triterpene act as an ERα antagonist (151). Another molecular mechanism was described in 2018 for ursolic acid and several other pentacyclic triterpenes consisting in the inhibition of the deubiquitinating protease USP7, thus promoting cell cycle arrest and apoptosis (152); through molecular docking the authors of the study revealed that ursolic acid binds to the ubiquitin pocket of USP7 through its C17-carboxyl group and C3-hydroxyl group. Therefore, ursolic acid can promote the discovery of new USP7 inhibitors with clinical efficacy in cancer therapy. Additionally, ursolic acid can strongly inhibit the *in vitro* growth of SKOV3 ovarian epithelial cancer cells as well as ovarian cancer stem-like cells which possess epithelial-mesenchymal transition properties; when administered in combination with cisplatin, ursolic acid caused an *in vitro* increase of cell sensitivity to cisplatin while *in vivo* significantly reduced ovarian tumors in a xenograft model on thymic BALB/c-nu nude mice (52).

An ursolic acid derivative, 15-oxoursolic acid, was isolated from the ethyl acetate fraction of the stem bark of *Rhododendron arboreum* Sm. extract and physicochemically characterized; its strongest *in vitro* antiproliferative effect was recorded against the human ovarian carcinoma (MDR-2780AD) cell line with low micromolar IC_50_ values (153).

Not all anticancer agents act by causing cytotoxicity; instead, other mechanisms may be involved in their antitumor overall effect. Such an example is the koetjapic acid, a seco-A-ring oleanane triterpenoid extracted from *Sandoricum koetjape* (Burm.f.) Merr., whose total extract was previously proven antiangiogenic; antiangiogenic agents inhibit the formation of new blood vessels and thus hinder tumor development (154). Despite its poor cytotoxicity, the koetjapic acid produced the dose-dependent inhibition of angiogenesis in an *ex vivo* experiment conducted on rat aorta by acting on the endothelial cell migration and differentiation and the VEGF expression, all major phases of the angiogenesis process.

We previously described asiatic acid, a pentacyclic triterpene isolated from *Centella* species, as an antiproliferative agent against breast cancer (see section *Breast Cancer*). In addition, asiatic acid was proven effective on SKOV-3 human metastatic ovarian cancer cells where it suppressed their migration and invasion ability (155). Asiatic acid induced elevated concentrations of epithelial markers (E-cad and KRT-7/14/19) and decreased amounts of mesenchymal markers (vimetin, N-cad and ZEB1/2) at mRNA and protein levels thus providing evidence for its inhibitory activity against the epithelial-to-mesenchymal transition and subsequently its anticancer potential in the management of ovarian cancer.

Three oleanolic acid glycosides (oleanolic acid-3-*O*-*β*-d-glucopyranosyl- (1→3)-*β*-d-glucopyranoside and oleanolic acid-3-*O*-*β*-d-glucopyranosyl-(1→3)-(2″-*O*-acetyl)-*β*-d-glucopyranoside) isolated from *Nematostylis anthophylla* (A.Rich. ex DC.) Baill. were investigated in terms of antiproliferative activity on the A2780 ovarian cancer cell line; a moderate but selective cytotoxic activity was found (156). Interestingly, the highest activity was noticed for acetylated compounds thus suggesting that an increased lipophilicity through acetylation may facilitate cellular uptake and anticancer efficacy. Oleanane-type triterpenoid saponins extracted from various parts of tea plants [*Camellia sinensis* (L.) Kuntze] were assessed in terms of *in vitro* antiproliferative activity on two platinum-resistant ovarian cancer cell lines OVCAR-3 and A2780/CP70; their strong cytotoxic effect was attributed to apoptosis induction *via* the extrinsic pathway as well as antiangiogenic properties by decreased levels of VEGF protein *via* the HIF-1α-dependent pathway (157). The synthetic oleanane derivative CDDO-Me (methyl-2-cyano-3, 12-dioxooleana-1, 9(11)-dien-28-oate) was found to induce apoptosis in OVCAR-3, OVCAR-5, SK-OV3, and MDAH-2774 ovarian cancer cells by increasing annexin V binding and cleaving the poly (ADP-ribose) polymerase (PARP-1) and procaspases 3, 8, and 9; also, CDDO-Me causes mitochondrial depolarization. Additional experiments showed that the proapoptotic activity exerted by CDDO-Me is accomplished *via* the inhibition of the AKT/NF-κB/mTOR signaling pathway (158). Gao et al. continued their investigations by assessing the role played by reactive oxygen species (ROS) in the antitumor activity of CDDO-Me against OVCAR-5 and MDAH-2774 ovarian cancer cells (111); the authors revealed that CDDO-Me has the ability to generate ROS (hydrogen peroxide, H_2_O_2_) and that pre-treatment with N-acetylcysteine not only inhibited ROS generation but prevented or even blocked all previously mentioned anticancer mechanisms induced by CDDO-Me, in particular the down-regulation of the AKT/NF-κB/mTOR signaling pathway. Therefore, the authors concluded that ROS play a pivotal role in the overall antitumor activity of CDDO-Me with mechanistic details further needing investigations.

The triterpenoid saponin oldhamianoside isolated from *Gypsophila oldhamiana* Miq., frequently used in Traditional Chinese Medicine, showed promising *in vitro* and *in vivo* results against human ovarian cancer (159); oldhamianoside inhibited SKOV3 ovarian cancer cells proliferation both *in vitro* and *in vivo*. *In vivo* studies on BALB/C nude mice revealed markedly suppressed plasma levels of TNF-α, IL-6, and MCP-1 as well as decreased expressions of VEGF, VEGFR2, caspase-3, and Bax/Bcl-2 ratio thus suggesting apoptosis induction and modulation of the inflammatory response and angiogenesis signal (159). Similar effects were reported for prostate cancer cells where oldhamianoside reversed the epithelial-mesenchymal transition by triggering E-cadherin induction and vimentin.N-cadherin suppression at MRNA and protein levels; in addition, oldhamianoside inhibited the β-catenin signaling pathway. Collectively, these data recommend the compound as potential anticancer agent for clinical use (160).

### Cervical Cancer

The polysaccharide and triterpenoid fractions, respectively, of the *Ganoderma lucidum* (Leyss.:Fr.) Karst extract were tested against a collection of cancer cell lines including cervix and breast cancer (161); both fractions showed efficacy and specificity at low concentrations on HeLa cervical cancer cells followed by MCF-7 breast cancer cells. Nuclear staining experiments revealed characteristic apoptotic cell morphological alterations, while the microtubule dynamic assay on HeLa cells indicated the specific inhibition of the microtubule network (161). *Ganoderma lingzhi*, a closely related strain to *G. lucidum* (Leyss.:Fr.) Karst, was the vegetal source of lucidumol C, an oxygenated lanostane derivative which was assessed against several cancer cell lines, including HeLa cells, and revealed remarkable cytotoxic properties and high selectivity on cancer cells (162). By comparing lucidumol C to its non-cytotoxic analogue, lucidumol A, the authors hypothesized that its cytotoxic activity is presumably related to the presence of a C-11 carbonyl group in its structure.

Also extracted from *G. lucidum*, ganoderic acid D is a tetracyclic lanostane derivative which inhibited HeLa cells proliferation with IC_50_ values of 17.3 ± 0.3 μM; the employed mechanism involves apoptosis and cell cycle arrest in the G2/M phase through the regulation of 21 protein expression. *In silico* assessment was used in order to uncover the interactions at molecular level between ganoderic acid D and targeted proteins; results showed that ganoderic acid is able to bind to the 14-3-3 protein family as supported further by further *in vitro* analysis. The 21 target proteins can fall into a single network either by direct protein-protein interactions or by the intervention of an intermediate molecule (163).

Taraxastane, (18alpha,19alpha,20beta)-ursane, was the subject of a very recent study published by Hu et al. who investigated its anticancer potential against DoTc2 human cervical cancer cells; the antiproliferative and selective concentration-dependent activity of taraxastane was reported, presumably through increasing ROS concentration and decreasing mitochondrial membrane potential (72). Unlike other previously studied pentacyclic triterpenes, taraxastane did not induce cell apoptosis but cell necrosis, as suggested by constant levels of Bax and Bcl-2 proteins; additionally, taraxastane blocked the JNK/MAPK signaling pathway by preventing JNK phosphorylation in a dose-dependent manner and inhibited cell migration and invasion, thus preventing metastasis.

The triterpene 3a,23-isopropylidenedioxyolean-12-en-27-oic acid (IPA) was isolated from *Aceriphyllum rossii* (Oliv.) Engl.; it increased the externalization of phosphatidylserine residues and the apoptotic DNA fragmentation in HeLa cervical cancer cells, activated certain caspases (–8,–9,–3) and caused the cleavage of the poly(ADP ribose) polymerase (PARP-1), induced mitochondrial membrane depolarization, increased Bax/Bcl-2 ratio and triggered endoplasmic reticulum stress (164).

An ursolic acid derivative, 23-hydroxyursolic acid, isolated from *Cussonia bancoensis* Aubrév. & Pellegr., inhibited the proliferation of HeLa cervical cancer cell in a dose-dependent manner; the in-depth study of the underlying molecular mechanisms revealed: 1) proteolytical fragmentation of caspases 3, 8, and 9; 2) markedly decreased expression of the anti-apoptotic protein Bcl-XL; and 3) apoptotic morphological alterations which can be inhibited by the Z-VAD-FMK [carbobenzoxy-valyl-alanyl-aspartyl-(O-methyl)- fluoromethylketone] pan-caspase inhibitor, thus indicating an apoptotic mechanism of cell death (165).

As previously stated, nanotechnology dramatically improved our ability to develop more effective alternatives in cancer treatment; ursolic acid was used as reduction agent for the preparation of gold nanoparticles. The resulting ursolic acid-capped gold nanoparticles were embedded in poly(DL-lactide-co-glycolide) (PLGA) and tested on cervical cancer cell lines CaSki, HeLa, C4-1, and SiHa; experimental data indicated a significant suppression of cervical cancer cell proliferation and metastasis tendency *via* apoptosis induction by caspase and p53 activation and anti-apoptosis-related signals suppression (138).

Based on the proven antitumor activity of the triterpenoid-rich extract of bamboo shavings, classified as functional food by the Chinese Health Ministry, its main component, friedelin, was assessed in terms of biological effects (166). Friedelin exhibited a strong antiproliferative activity against four cancer cell lines including HeLa cells, in a time- and dose-dependent manner, thus suggesting that the extract effect can be at least in part attributed to its presence.

A mixture as well as isolated oleanane saponins from *Anemone flaccida* Fr. Schmidt were tested in terms of anticancer molecular mechanisms on human cancer cell lines, including HeLa cervical cancer (167). The authors reported that the COX-2/PGE2 signaling pathway is involved in the apoptotic mechanism of cell death induced by the oleanane saponins, as supported by their higher efficiency in cells with overexpressed COX-2. In addition, lower IC_50_ values indicating stronger apoptotic effects compared to other pentacyclic triterpenoids were reported for oleanane saponins, effects attributed to the polarity of the C3-glycoside moiety.

A representative of the tetracyclic triterpene group, cucurbitacin E (*Cucumic melo* L.), was assessed *in vitro* on two cervical cancer cell lines HeLa and CaSki and revealed increased apoptotic processes and irreversible cell cycle arrest in the G2/M phase as well as the up-regulation of the death receptor DR5 in a dose-dependent manner; no necrotic events were reported (168). Another tetracyclic triterpene, cucurbitacin IIb (23,24-dihydrocucurbitacin F) isolated from the methanolic extract of *Ibervillea sonorae* (S. Watson) Greene pronouncedly inhibited the proliferation of HeLa cells and induced apoptotic cell death in a selective manner (169).

Two natural saponin fractions of the *Securidaca longipedunculata* Fresen. bioactivity-guided methanolic extract, containing triterpenoid glycosides, were biologically evaluated through multiple assays conducted *in vitro* on two cervical cancer cell lines, CaSki and Bu25TK (170); an antiproliferative activity was reported for both fractions with IC_50_ values within the low micromolar range. Late and early apoptotic processes were recorded for the two fractions, respectively, achieved through the inhibition of the antiapoptotic proteins MCL-1 and BCL2L1 and low expressions of AKT-3 and VEGFA. Thus, one can state that the two saponin fractions inhibit the antiapoptotic (PI3K)-AKT/mTOR/NF-kB dependent signaling pathway.

Pentacyclic triterpenes with a polyhydroxylated A ring were found to exhibit important biological activities with a potency in direct dependency to the number of hydroxyl groups; this is presumably due to the involvement of the hydroxyl group in the antitumor and anti-inflammatory effect of the respective pentacyclic compound (171). (1β, 2α, 3β, 19β, 23)-1,2,3,19,23-pentahydroxyolean-12-en-28-oic acid is a new phytochemical compound isolated from *Euphorbia sieboldiana* C.Morren & Decne., identified as a strong inhibitor of cervical cancer cells proliferation in a dose- and time-dependent manner; it exerts a selective cytotoxic activity by inducing apoptosis and cell cycle arrest in the G1 phase by inactivating the TNF-α–TAK1–IKK-NF-κB axis and TNF-α–stimulated NF-κB activity and subsequently down-regulating the NF-κB target genes. The newly discovered compounds also showed anti-metastasis activity on HeLa cells and further studies revealed that the NF-κB signaling cascade was decreased *via* ROS generation (171).

Semisynthetic derivatives bearing cyano-enone moiety at A ring level such as the previously oleanane derivative CDDO are considered promising new agents against malignant tumors; a new glycyrrhetinic acid derivative soloxolone methyl was assessed on human cervical carcinoma KB-3-1 cells by a transcriptomic approach (172). The results clearly indicated the stress of the endoplasmic reticulum through several transcriptional regulators; in addition, the compound modulates the expression of key genes involved in the proliferation of cervical cancer cells by binding to their active sites as suggested by molecular docking.

Lupeol is a lupane-type pentacyclic triterpene found in many foods of vegetal origin and which exhibits selective antitumor properties; Prasad et al. investigated the effects exerted by lupeol in HeLa cervical cancer cells and reported its inhibitory activity on cell proliferation and viability by inducing S-phase cell cycle arrest and apoptosis through increased expressions of the apoptotic markers (cleaved PARP, Bax/Bcl2 ratio). In addition, lupeol triggers mitochondrial superoxide generation followed by a decreased healthy mitochondrial mass in cancer cells (173).

### Prostate Cancer

Prostate cancer is characterized by a slow progression and long latency; therefore, the addition of phytochemicals with therapeutic and/or prophylactic properties to consecrated drug therapies may provide the means to control both the disease progression and its mortality rate (174).

Based on the finding that metformin, which activates 5′-AMP-activated protein kinase (AMPK) in diabetic patients, simultaneously induces a decrease of cancer occurrence, Akhtar et al. investigated in 2015 the extract of *Ficus microcarpa* L.f. in order to identify metformin mimetic compounds with anticancer potential (175). They isolated the pentacyclic triterpenoid plectranthoic acid that far exceeded metformin in terms of AMPK activating properties; when applied on PCa prostate cancer cells, plectranthoic acid inhibited cell proliferation and disrupted cell cycle in G0/G1 phase by up-regulating cyclin kinase inhibitors (p21/CIP1, p27/KIP1) as well as induced apoptosis by the suppression of the mTOR/S6K signaling pathway. While the apoptosis induction is dependent on AMPK, the autophagy of PCa cells occurs by different but still unknown mechanisms; moreover, the association of metformin inhibits the biological effects of plectranthoic acid. The cytotoxic effects of aqueous and ethanolic extracts from *Ficus religiosa* L. bark were also reported in SiHa and HeLa cervical cancer cell lines. The mechanisms underlying the anticancer activity in SiHa cell lines involved the inhibition of cell cycle progression in G1/S phase correlated with an increase of p53, p21 and pRb protein expression (176). In both SiHa and HeLa cell lines, were the *Ficus religiosa* L. extract induced apoptosis, a substantial decrease of viral oncoproteins E6 and E7 expression was reported (176).

Lupeol was found active against androgen-sensitive human prostate cancer as suggested by *in vitro* studies conducted on LNCaP and CWR22Rr1 cells (177). Its antiproliferative and apoptotic effects manifested in a dose-dependent manner by activating the Fas receptor–mediated apoptotic pathway; the combined cell treatment with lupeol and anti-Fas monoclonal antibody led not only to an additive effect but a synergistic one. The *in vitro* experimental data were confirmed by further *in vivo* studies on prostate cancer models developed on athymic nude mice where lupeol significantly reduced both tumor growth and PSA (prostate specific antigen) plasma levels.

Cucurbitacin E was previously mentioned as active compound against cervical cancer (see section *Cervical Cancer*); Dong et al. documented its inhibitory activity on human prostate tumor growth (178). Cucurbitacin E significantly inhibited the *in vitro* proliferation, migration and tubulogenesis of human umbilical vascular endothelial cells by inducing apoptosis, blocked the *ex vivo* angiogenesis process and suppressed prostate tumor growth in a murine model. In-depth studies of the underlying molecular mechanisms revealed that the antiangiogenic effect was achieved through the inhibition of the VEGFR2-mediated Jak-STAT3 and mitogen-activated protein kinases signaling pathways (178). Another study aiming to decipher the anticancer mechanisms of cucurbitacin type compounds assessed the antiproliferative effect of 23,24-dihydrocucurbitacin F, isolated from the root of *Hemsleya amabilis* Diels, on three prostate cancer cell lines (DU145, PC-3, and LNCaP). The study concluded that the anti-proliferative activity of the compound was a result of cytokinesis failure, cell cycle arrest at G2/M phase, cell growth inhibition and apoptosis due to upregulation of p21CIP1, downregulation of cyclin A, actin aggregation and cofilin-actin rod formation (179). The same growth inhibition, attributed to cell cycle arrest at G2/M phase and apoptotic cell death was described in SKBR-3 and MCF-7 breast cancer cell lines upon treatment with cucurbitacin B extracted from *Trichosanthes cucumerina* Linn (180, 181). The mechanism involves the depletion of the nuclear β-catenin and galectin-3 and the interruption of Wnt signaling pathway (180, 181).

The semisynthetic oleanane derivative CDDO-Me was assessed in terms of efficacy in the prevention of prostate cancer development and progression on a murine model of transgenic adenocarcinoma (158). Both early and delayed administration of CDDO-Me to experimental mice was able to inhibit the development of prostate adenocarcinoma and its distant metastasis; *in vitro* studies on TRAMPC-1 prostate cancer cells revealed the inhibition of the antiapoptotic p-AKT, p-mTOR and NFκB proteins thus indicating AKT as a potential *in vivo* target for CDDO-Me. The compound was also investigated on the hormone-refractory PC-3 (AR^-^) and C4-2 (AR^+^) prostate cancer cell lines where it strongly inhibited cell proliferation and induced apoptosis, when applied in very low concentrations; in terms of molecular mechanism, CDDO-Me inhibited p-AKT, mTOR and NF-kB signaling proteins and, consequently, their downstream targets. Further *in vivo* studies on PC-3 xenografted nude mice showed a reduction in tumor growth (182). More in-depth studies have shown that CDDO-Me directly inhibits the AKT kinase activity both *in vitro* and *in vivo* without affecting the upstream kinase PDK1 which activates AKT through phosphorylation. The AKT inhibition by CDDO-Me led to the inactivation of downstream proapoptotic proteins and was not connected to the activity of phosphatases involved in AKT-dephosphorylation (183). The proapoptotic mechanism of CDDO-Me was further detailed by the same group who investigated the role of ROS in the above-mentioned process by using the LNCaP and PC-3 prostate cancer cell lines; the authors concluded that ROS are generated from mitochondrial and non-mitochondrial sources and are closely related to the apoptosis induction, as emphasized by its endogenous or exogenous inhibition which prevents CDDO-Me-induced apoptosis (184). The effect of the human telomerase reverse transcriptase (hTERT) in mediating CDDO-Me anticancer activity was studied *in vitro* and *in vivo* on the above mentioned LNCaP and PC-3 prostate cancer cell lines by the same research group; the study revealed that CDDO-Me antiproliferative and apoptotic effects were closely related to the inhibition of hTERT gene expression, its telomerase activity and the regulation of hTERT modulating proteins. Similar effects were recorded *in vivo* on transgenic mice bearing xenografted prostate cancer thus emphasizing hTERT as a potential target for CDDO-Me in prostate cancer (185).

A series of natural and semisynthetic triterpenoids were assessed as antiproliferative and antimetastatic agents in prostate cancer; lupeol, calenduladiol and helantriol B2 isolated from *Chuquiraga erinacea* D.Don were the natural triterpenoids involved in the study while 19 derivatives were prepared by the chemical modulation of lupeol and calenduladiol (186). The entire group of compounds was tested on PC-3 and LNCaP prostate cancer cells; sulfate derivatives were emphasized as the most potent antiproliferative agents with one compound, calenduladiol-3β-monosulfate, exhibiting anti-migration effects on PC-3 cells. In addition, heliantriol B2 and 3β-aminolupane produced antimigratory effects on LNCap cells in a dose-dependent manner.

Two new oleanane-type triterpenoid compounds were isolated from *Gladiolus segetum* Ker-Gawl. corms and their structures were established and confirmed through physicochemical analysis (187) their *in vitro* investigation on five different human cancer cell lines revealed strong cytotoxic activities in particular against PC-3 prostate cancer cells. Molecular docking studies indicated the inhibition of the HER-2 enzyme as the potential underlying mechanism of the cytotoxic effect.

Marine triterpene glycoside were previously mentioned as effective antitumor agents in various cancers (see section *Breast Cancer*); a representative of this group is frondoside A which exerts a highly selective and strong antiproliferative activity against castration-resistant prostate cancer cell lines PC-3 and DU145, including cells that became resistant to enzalutamide and abiraterone (188). Frondoside A acted by inducing cell cycle arrest and caspase-dependent and independent apoptosis by up-regulating pro-apoptotic proteins and down-regulating anti-apoptotic ones; in addition, the compound interferes with the regulation of proteins engaged in tumor invasion and metastasis and inhibits the pro-survival autophagy. Its antimetastatic activity was verified *in vivo* on NOD SCID mice where frondoside A not only inhibited tumor growth but also lowered the occurrence of lung metastasis without causing significant organ toxicity; an immune modulating effect was noticed as well.

Celastrol is an active anticancer triterpenoid isolated from the Chinese herb *Tripterygium wilfordii* Hook. f. which was tested by Ji et al. in 2015 on DU145 prostate cancer cell line in various concentrations; the compound exhibited an antiproliferative effect in a time- and dose-dependent manner by inducing apoptosis and G0/G1 cell cycle arrest; the authors found an overexpression of the hERG channel in the prostate cancer cells downregulated by celastrol in a dose-dependent manner at protein and mRNA level (189). Despite its potent antitumor effects, celastrol is characterized by poor physicochemical and pharmacokinetic properties which limit its clinical use; this unfavorable profile can be improved through nanotechnology. Thus, biocompatible poly(ϵ-caprolactone) nanoparticles were prepared and loaded with celastrol by using a nanoprecipitation method; their antiproliferative activity was tested on LNCaP, DU-145, and PC3 prostate cancer cell lines which were inhibited in a dose-dependent manner with IC50 values below 2 μM. An increase in cytotoxicity was noticed for the encapsulated celastrol compared to the pure drug manifested through apoptosis and cell cycle arrest (190).

Triterpene oleanane saponosides have been suggested as safe and tolerated therapeutic agents in advanced prostate cancer; desglucoanagalloside B was isolated from *Lysimachia ciliata* L. and tested on human prostate cancer cells obtained from brain (DU-145) and bone (PC-3) metastasis. Sub-micromolar concentration of saponine induced apoptosis and inhibited cell proliferation by releasing cytochrome C and activating caspase 3/7. Based on the knowledge that saponins’ cytotoxic effects are attributable to both the saccharide moieties and the aglycone, the activity of desglucoanagalloside B was compared to that of the closely related anagallosaponin IV which bears an identical oligosaccharide side chain; anagallosaponin IV revealed low cytotoxic and proapoptotic effects thus emphasizing the specific action of desglucoanagalloside B which also exhibits high selectivity against cancer cells. Additionally, only desglucoanagalloside B was able to diminish cell invasion potential by affecting cell elastic properties (191). A mixture of the two compounds was assessed for its chemosensitizing properties on the same prostate cancer cell lines; the mixture was used in low concentrations that were not able to induce cytotoxic or cytostatic effects. However, when combined with mitoxantrone, a synergistic highly selective cytostatic, proapoptotic and antimetastatic activity was recorded (192). Another triterpene saponin, nummularoside, extracted from *Lysimachia nummularia* L. was assessed by the same research group displaying significant anticancer selective properties against DU145 and PC-3 prostate cancer cells (193). Afrocylamin A, also an oleanane-type triterpene saponin isolated from *Androsace umbellata* (Lour.) Merr., caused cytotoxic and apoptotic effects on DU145 prostate cancer cells by inducing sub-G0/G1 cell cycle arrest and autophagic cell death through the inhibition of the PI3K/AKT/mTOR signaling pathway. In addition, afrocylamin A exhibited antimetastatic effects manifested in a time- and concentration-dependent manner; all *in vitro* activities were supported and confirmed by *in vivo* results on a DU145 xenografted model in mice (194).

The *Ganoderma lucidum* (Leyss.:Fr.) Karst extract in high doses exerted inhibitory effects on DU-145 prostate cancer cells viability, migration, and invasion by inducing apoptosis in a dose- and time-dependent manner; the authors indicated as potential mechanism the regulation of matrix metalloproteases (195). Another research group exposed four prostate cancer cell lines (LNCaP, 22Rv1, PC-3, DU-145) and one normal prostatic epithelial cell line (BPH) to the total triterpenoid extract of *G. lucidum* used in different doses and time intervals; apoptosis and G1-phase cell cycle arrest in a dose-dependent manner were identified as potential mechanisms for its anticancer overall effect (196). The group of Liu et al. showed that two lanostane-type triterpenoids, ganoderic acid and 5a-lanosta-7,9(11),24-triene15a,26-dihydroxy-3-one, isolated from *Ganoderma lucidum* (Leyss.:Fr.) Karst present an inhibitory effect on the activity of 5α-reductase with an IC_50_ of 10.6 µM and 41.9 µM (197). However, due to its role in testosterone conversion into dihydrotestosterone, 5α-reductase and its inhibition remains a controversy in prostate cancer prevention and treatment.

Corosolic acid is a natural ursolic acid derivative isolated from plants belonging to the *Rosaceae* and *Lamiaceae* families which show antitumor, antiproliferative, and apoptotic/non-apoptotic effects against numerous human cancer cell lines through various molecular mechanisms and low toxicity. Despite the fact that there are no available structure-activity relationship studies, based on its structural resemblance to ursolic acid one may assume that the cytotoxic activity is related to the C3-hydroxyl group and the C28-carboxyl group in its molecule (198). Corosolic acid was proven able to inhibit cell growth and induce apoptosis in PC-3 and DU145 human prostate cancer cell lines thus showing potential for the therapy of castration-resistant prostate cancer; it activated two pro-apoptotic signaling pathways associated with endoplasmic reticulum stress as suggested by increased levels of specific endoplasmic reticulum stress markers such as IRE-1/ASK1/JNK and PERK/eIF2α/ATF4/CHOP finally leading to AKT inactivation and cell death (199).

Two dammarane-type triterpenes were isolated from the aerial parts of *Cleome khorassanica* Bunge & Bien ex Boiss. and tested on Du145 and LNCaP prostate cancer cells; in terms of cytotoxicity, the two new compounds inhibited cell growth in a dose-dependent manner. One compound (20,25-dihydroxy-3-oxodammarane triterpene) was more selective on the androstane-independent DU145 cell line while the other (3-oxo-4-oxa-A-homo-25,26,27-trinordammarano-24,20-lactone triterpene) targeted the androstane-dependent LNCaP cell line (53). Another dammarane-type triterpene is gypensapogenin H which was isolated from the total extract of *Gynostemma pentaphyllum* (Thunb.) Makino and, when tested on DU145 and 22Rv1 prostate cancer cells, inhibited proliferation and cell survival by inducing G1 cell cycle arrest and apoptosis; simultaneously, the compound almost lacked antiproliferative effects on a series of healthy cell lines (gastric, kidney, fibroblast) thus showing a selective antitumor activity (52).

## Closing Remarks and Future Perspectives

Like the universe itself, the world of known natural compounds with definite and selective anticancer properties is continuously expanding. Triterpenes are a class of secondary metabolites which occur in a wide variety of plant species, including common dietary plants like apples and olives, and exhibit a large plethora of biological activities. Their anticancer and chemopreventive effects have been investigated extensively partly revealing their underlying molecular mechanisms; yet some of these mechanisms are still to be uncovered. Basically, their anticancer mechanism consists in inflammation and oxidative stress inhibition, cell cycle regulation and apoptosis induction as well as antiproliferative effects. Very remarkable is the fact that triterpenes are able to simultaneously modulate multiple signaling pathways thus limiting the ability of tumor cells to involve different survival pathways in order to continue to grow.

A large body of evidence indicates that triterpenes induce selective anticancer effects in numerous cancers, including reproductive hormone-dependent types; it is interesting to notice that one compound is able to produce similar effects in different types of cancer. Many studies have been conducted on isolated compounds; however, the evaluation of phytochemical mixtures emphasized a potential synergistic anticancer effect; also, triterpenes used in combination with conventional chemo or radiotherapies may increase their efficacy and attenuate their side effects thus making the therapy more bearable for cancer patients and increasing their compliance.

The unfavorable pharmacokinetic profile of triterpenoids due to their poor water solubility can be overcome by the design and preparation of semisynthetic derivatives with higher pharmacological efficacy and lower toxicity; some of these derivatives are already subjected to clinical trials. A second attractive option is the use of nanotechnology which allows the preparation of complex nanoformulations with improved pharmacological profiles. Various nanoparticulated systems were designed and tested such as liposomes, nanocapsules, colloids, micelles, etc.

However, the work of researchers never stops; a considerable amount of efforts must still be employed in the field of triterpenes and their biomedical use; an important aspect is the development of specific and reliable biomarkers in order to be able to assess the clinical outcome; also, *in vivo* results must be supported by long-term epidemiological studies as well as larger cohorts clinical studies. Nevertheless, triterpenoids have been documented so far as selective and promising candidates in the design of chemopreventive and therapeutic strategies against malignant diseases including hormone-dependent cancers.

## Author Contributions

CŞ, MV—designed the concept of the review and participated in manuscript writing and editing. RG—collected literature data, participated in manuscript writing and formatting. CD, RR, CT, O-JR, GN, MM—equally participated in manuscript writing. AM—participated in manuscript writing, proof editing, graphical design, and supervised the entire process. All authors contributed to the article and approved the submitted version.

## Funding

This work was supported by an Internal grant at “Victor Babes” University of Medicine and Pharmacy, Grant 1EXP/1233/30.01.2020 LUPSKINPATH, Project Manager: CŞ.

## Conflict of Interest

The authors declare that the research was conducted in the absence of any commercial or financial relationships that could be construed as a potential conflict of interest.

## Abbreviations

AKT, protein kinase B; AMPK, 5′-AMP-activated protein kinase; Apaf-1, apoptotic protease activating factor 1; Bcl-2, B-cell lymphoma 2; Bax, Bcl-2 associated protein X; Cdc2, cell division control protein, cyclin-dependent kinase; Cdc25C, cell division cycle 25C; CDK, cyclin-dependent kinase; COX-2, cytochrome c oxidase subunit 2; CXCL12, C-X-C motif chemokine ligand 12; ER, estrogen receptor; ERK, extracellular regulated MAP kinase; hTERT, human telomerase reverse transcriptase; JNK, c-Jun NH2-terminal kinase; MAPK, mitogen-activated protein kinases; MMP, mitochondrial membrane potential; MPTP, mitochondrial permeability transition pore; mTOR, mammalian target of rapamycin; NF-kB, nuclear factor kappa-B; PTHrP, parathyroid hormone-related protein; PCa, prostate cancer cells; PGE2, prostaglandin E2; PI3K, phosphoinositide 3-kinase; PLGA, poly(DL-lactide-co-glycolide); ROS, reactive oxygen species; STAT3, Signal transducer and activator of transcription 3; SERM, selective estrogen receptor modulator; USP7, ubiquitin-specific-processing protease 7; VEGF, vascular endothelial growth factor.
